# Recent Trials in Lunacy
*1. Roberts *v*. Kerslake. Tried at the Summer Assizes, Warwick, 1854.2. The Duke of Manchester *v*. Bennett. Tried at the Spring Assizes, Kingston, 1854.3. Queen *v*. Brough. Tried at Guildford, August 9, 1854.


**Published:** 1854-10-01

**Authors:** 


### Art. VII.—RECENT TRIALS IN LUNACY.*

We cannot permit our present number to be published without bringing specially under the notice of our readers three important trials, involving the plea of insanity in criminal and civil cases, that have occurred within a recent period. The two civil cases will no doubt be carefully read and well studied by all who take an interest in medico-legal questions. The criminal case has, from its peculiar associations, which it is unnecessary for us further to allude to, excited an unusual degree of professional and public interest, and has given rise to much animadversion and discussion in the public and medical journals of the day. This case will, from its peculiar features, take a prominent position among the *Causes Célèbres* of British Criminal Jurisprudence. We will proceed, in the first place, to the consideration of the two civil cases.

Roberts *v.* Kerslake.—In this case Elizabeth Roberts was the plaintiff, and Charles Edwin Kerslake, and Mary Ann, his wife, were the defendants. It was an issue from the Court of Chancery, in which the plaintiff affirmed that Henry Roberts duly made and executed his last will and testament on the 4th Dec. 1853, which was denied by the defendants. This case was tried at the Warwick Summer Assizes, before Mr. Baron Parke and a special jury, in August last. The facts of the case are briefly as follows:—Mr. Roberts, whose will was disputed on the ground of the insanity of the testator, had for many years carried on the business of a grocer and general dealer in the towns of Warwick and Leamington. In 1849 he retired from business, after having amassed a considerable fortune. He made his will entirely in favour of his wife, on the 4th Dec. 1853. He died on the 1st Feb. 1854. It appears that he had no other near relations, excepting a sister, who had married a gentleman of the name of Kerslake, who was heiress at law, and who now sought to dispute the will. Mr. Roberts married in 1835, and, according to the Attorney-General's statement, there had existed during their married life the greatest cordiality and affection. The question really at issue was, whether, on the 4th Dec.

* 1. Roberts *v.* Kerslake. Tried at the Summer Assizes, Warwick, 1854.
2. The Duke of Manchester *v.* Bennett. Tried at the Spring Assizes, Kingston, 1854.
3. Queen *v.* Brough. Tried at Guildford, August 9, 1854.

1853, the day upon which he made his will, he was of sound and disposing intellect.

The following evidence was laid before the jury on behalf of the plaintiff:—

*Mr. Wm. Osborn* had been mixed up with him in several public matters, and seen him frequently. He was a shrewd man, and always took care of his own interest. He was occasionally odd-tempered, passionate, and strange in his manner. Not more so of late, that he was aware of.

*Mr. Wm. Satchell* said Mr. Roberts was excitable, and liked to have his own way. Always thought him peculiar. Met him as late as October or November last, and *thought he saw a great change in his bodily condition. Saw no change in his mind.* At public meetings *he was loud and excited.* He became a candidate for Warwick in 1847, and had some small copper coins on which were struck the words, "Vote for Roberts."

*Mr. David Johnson* had met Mr. Roberts at the local Board. What he said was consistent, but his manner was inconsistent. What he said was very sensible. Never saw anything to the contrary of his being sane and rational. No change struck him in his general demeanour or conduct, public or private, up to the last time he saw him.

*Mr. Joseph Fletcher* had never perceived anything the matter with his mind. Had seen him excited.

*Mr. Thomas Gibbs* saw him on the 12th October. His understanding was very good. He was a good man of business.

*Mr. Wm. Eyres* last saw him on the 26th of October. Up to that time he had noticed no failure in his mind, but he became thinner. Considered he was a man of sound understanding.

*Mr. H. Elliot Hoole* had had business transactions with him. Received a visit from him in September, and paid him a visit in October. Did not observe much difference in his health in September. He had always been ill since he knew him. *He spoke to him about having put up for Warwick.*

*Mr. Richard Archer Wallington:* The first allusion made to the settlement of his affairs by Mr. Roberts was eighteen months previously. He said, if anything happened to him, he wished his wife to send for him immediately, as he wished him to have the arrangement of his affairs. Was in the habit of seeing him frequently. He said that his sister had influenced the father in making the will. Transacted business with Mr. Roberts on the 18th of November, 1853, and on Monday, the 28th November, and there was not the least failure in Mr. Roberts's mental capacity that he could discern. He had changed in bodily health twelve months previously. The following conversation took place at the interview on the 28th November:—"I said, 'Mr. Roberts, have you made your will?' He said, 'No; he thought of making one.' I then referred to the conversation eighteen months before, and told him it was useless to send for me, unless he had already prepared what is to be carried out. His reply was, 'I mean to come to you, and you shall make the will.' I said, 'How do you wish to

Q Q 2

dispose of your property?' He said, 'I shall leave the whole to my wife; and those who are good to her, she can be good to them.' I said, 'This is a very simple affair, and you had better carry it out at once.' He said, 'I don't know about the witnesses; I don't wish it to be known I have made a will.' I said, 'You can come to our office, and either my partner or my clerk can witness it with me.' He said, 'That would not do; it would be talked of.' After further conversation, he said he thought Dr. Franklin would witness it. I said, 'You can't have a better man; let me speak to him about it.' He said, 'I will speak to him, and you can come up some time when he is here.' I said, 'Don't delay this matter, as you have made up your mind about it.' I then said, 'Are you aware that if anything were to happen to you, your wife would not have sixpence of your property?' He appeared astonished, and said the matter should be done." Was to speak to Dr. Franklin on the subject. On Thursday or Friday he saw Dr. Franklin, and asked if Roberts had spoken to him. Inquired of Dr. Franklin the state of Mr. Roberts's health, and in consequence of what Dr. Franklin said, he wrote to Mrs. Roberts to tell her Dr. Franklin's opinion, on the Thursday or Friday. Saw Mr. Roberts again on Friday, Dec. 2nd, at his house, on other business. Considered him worse in health, but he was quite rational in conversation. He saw him on *Sunday* morning, before church service. Conversed with him for five or six minutes, but did not speak to him on business. Showed not the slightest symptom of mental excitement; on the contrary, was then calmer than he had seen him for years. Was expecting to leave for London on the following morning, and in consequence of that, and what he heard *from Dr. Franklin*, he determined to call on Mr. Roberts again the same Sunday. Called about four o'clock, and he was then out for a walk, and came in by himself. *He was quite free from excitement in the afternoon*, but he appeared stronger, as if, as he said, refreshed from his walk. *Asked Roberts whether he had altered his intention with respect to his will, and he said he had not.* Said, '*I think you had much better make the will to-day, as Dr. Franklin and myself are both at liberty.*' *He showed some disinclination at first*, and said *he would do it in the course of the week.* I said, 'I cannot make it for you then, for I expect to leave for London to-morrow, and may be detained a week or a fortnight; *you had better ring the bell, and send for Dr. Franklin.* Dr. Franklin was sent for. I made the will. Wrote it while Dr. Franklin was being sent for. *Asked Mr. Roberts if he had altered his intention with respect to the disposal of his property.* Wrote the will in Mr. Roberts's presence, *and read it over before Dr. Franklin arrived.* When *the Doctor* arrived, I told him Mr. Roberts was desirous of making his will. Handed the will to Mr. Roberts, and said, 'Read it yourself.' Mr. Roberts read and signed the will. I mentioned the usual formal publication, which words he repeated after me. Dr. Franklin said, 'Mr. Roberts, you quite understand the nature of your will?' Mr. Roberts said he did. They then put their signatures to it as attesting witnesses to the will. I asked Roberts whether I should take the will with me or leave it; and he told me to take it with me. After the signature of the will, remained with him about ten minutes. He was in the same state when I left him as during the remainder of the interview. He was perfectly competent to dispose of his property, or I would not have signed it. I had latterly seen him under much depression. He was less depressed in his spirits on the Sunday when he signed his will, than I had seen him for a long time. He was calm and collected, and free from despondency. He gave no reason for wishing to put off making his will. I considered it was mere matter of procrastination.

*Mr. Wm. Smith:* On Saturday evening, the 3rd of December, went to see Mr. Roberts at his house. Dr. Franklin was there. Asked how he was, and *he put his hand up to his head, complaining bitterly of pain.* Dr. Franklin said he had been endeavouring to prevail npon him to take medicine, and he again asked him to take some. He refused, saying, "*My life is valuable for my wife's sake.*" *He gave the same answer several times over to Dr. Franklin and himself.*

*Mrs. Roberts,* the widow, deposed that Mr. Roberts's health declined in 1850. He wasted considerably within the last few months. *He was lately more excited in mind;* he suffered a great deal from sciatica; that made him irritable. Although he became more irritable, she found no change in his understanding with reference to matters of business,—not the least. On the Saturday, December 3rd, she sent for Mrs. Kerslake, his sister, stating that her brother was very ill, and she should like to see her in consequence. She came between ten and eleven o'clock, and saw her brother. She showed her Mr. Wallington's note. Mrs. Kerslake said she was not aware her brother was so ill. She told her he was very ill; and Mrs. Kerslake said she thought her brother ought to settle his affairs. She had heard that Mr. Roberts and Mr. Wallington had had some conversation about a will. She did not know how her husband intended to dispose of his property. When Mrs. Kerslake left, Mrs. Roberts accompanied her. When he came back, he said he would go to the Public Hall and read the papers. *When he came back from there, there was something peculiar about him.* He had met with Mr. Payton and Mr. Cookes. He said, "That fellow Payton wanted to come in and take wine; but I would not have it." *He was very much excited, and very different to when he went out. Dr. Franklin came soon afterwards, and wanted to induce him to take the medicines which he had prescribed for him the day previously. He refused.* He never would take medicine if he could avoid it. He had no objection to external applications. He would apply to medical gentlemen for advice, but he would not take their medicines. On this occasion he said, "No, Dr. Franklin, *I will not take it; my life is too valuable to this good lady, my wife.*" He was in the habit of expressing himself strongly, and was very obstinate at times. The excitement lasted half-an-hour. He went to bed, and passed a good night. Two men were sent by Dr Franklin, but she did not know that till the next morning. Tilsley, the gardener, stayed in the house that night, but she did not know it till after. He once *imagined there were people outside the house. That was, perhaps, twelve months previously. She thinks he called two policemen out of*

*the window, to ask them to look round.* It was the horse that made the noise. *She recollects his getting up one night in November, and burning some papers.* He said he had some old papers, and should like to destroy them. She got up too. They were old letters and town papers. She thinks it was between midnight and break of day. He was a bad sleeper. He always formed his business plans at night, and would get up and make a note of them. He would sometimes get up and write letters in the night. This had been the case for many years. On Saturday he passed a good night, and on Sunday morning he appeared better. Previously to that his nights had been bad, and this was the first good night he had had for some time. After dinner, he walked out for about two hours. When he returned, he said he was much better, and refreshed. Mr. Wallington came and waited till Mr. Roberts came in. When he returned, Mr. Wallington asked for a sheet of paper, on which she had understood the will was to be made. She was, that day, constantly in communication with her husband. There was nothing in his conduct that she perceived, that gave any indication of his mind being affected,—not the slightest. He was calm that day, and there were no signs of excitement. He did not talk a great deal. He was not in his usual high flow of spirits. His spirits were lower towards evening, and he complained of his head. He was excited next day. On Monday morning, Mr. Roberts appeared to be going on favourably. In about an hour, before the breakfast things had been removed, he said to his cousin, who had come in, "*Selina, come out of the room; the chimney is on fire.*" There was a very large fire, and they were burning Cannel coal in a stove, which made a great noise. She has been alarmed by the same thing since then. Her husband went out of the room. The fire was put out. He was very excited in the morning, and was worse that day. Dr. Franklin came, and could not succeed in inducing him to take medicine.

*Joseph Franklin* had observed his health give way for two or three years. Saw him two or three times in the week previous to Sunday, the 4th December, and thought him ill. Went to his house one evening that week. His intellect was as usual. Saw him about twelve o'clock on Sunday, the 4th. Mrs. Roberts was with him. He was very self-possessed and composed,—more so than he had seen him that week. He seemed more subdued and calmer than when in his usual state of health. He was naturally an excitable man. Never found him very violent, but boisterous. Never saw him in any very violent passion; he liked to have his own way, was self-willed and obstinate. On this Sunday he was perfectly calm. His state of mind was calmer that night than usual. Had never seen him so calm or self-possessed before that night. His ordinary manner was very cheerful, very talkative, very merry, and he liked to have most of the conversation to himself, expressing himself very strongly, with a loud voice. He was not in that state on the Sunday. He understood and answered all the questions he put to him. Considered him as capable of understanding business as ever.

*Mr. Jones* was driving to Weston on Sunday, the 4th, and met

Mr. Roberts. There was nothing remarkable in his manner. He appeared perfectly calm.

*Mr. Chattaway* saw him on Sunday, the 4th December. Previous to that had noticed his health declining for some time. He never complained to him till that day. Went there between five and six in the evening, and said, "I am sorry to hear that you are so unwell." He said, "I am ill." Stopped with him a quarter or half-an-hour. They conversed the whole time on various topics. There was nothing to induce him to believe that his intellect was affected. He was particularly calm,—more so than I had ever seen him. He was for some time before generally in high spirits, but irritable to some persons. There were certainly no symptoms of want of clearness in his mind. He was capable of transacting any business.

*Mr. Alexander* had known him well for twenty years. Saw him last on the 5th December, when he conversed with him rationally for an hour and a half upon town affairs.

*William Alderton*, the attendant sent by Dr. Conolly, was the next witness examined. His evidence will be found detailed in a subsequent part of this article.

*Dr. Franklin* deposed that he first knew Mr. Roberts in 1852, when he attended Mrs. Roberts through a long illness. Attended him prior to his death. His attendance upon him commenced on the 2nd December. Prescribed for him in 1852. He was then suffering from neuralgia, arising from the state of his liver. *On Friday, the 2nd of December last, he was called in, and found him nervous, feverish, excitable, and with more or less heat about him. Then attributed the whole of his indisposition to a congested state of the liver, and enlargement of the liver.* That state of the liver was a condition likely to act on the brain, and to produce mental as well as bodily excitement. It was in the afternoon he saw him. *Saw him again on the following morning.* Prescribed for him, but could not induce him to take medicine. On the Saturday morning he was in pretty much the same state. Again urged him to take medicine, but without success. Saw him again on Saturday evening, and he was more excited and heated. Thought him worse. *He thought it necessary to take the precaution of having two men in the house.* He was in that excited state, that he thought himself responsible. *The state of his liver was such as was likely to produce delirium. Urged him to take medicine that evening. He resisted all his efforts to persuade him, and he could not succeed. He said his life was too valuable for his wife.* He had recovered considerably before he left, but he thought some slight delusion had set in. He could not tell whether what he said arose from delusion or trick to evade taking the medicine. That was the only delusion he noticed, if delusion it were. Saw Mr. Roberts about ten on Sunday morning. He had had a good night, and was much improved. He was quite composed. There were no remains of excitement or delusion. He appeared to be in possession of his faculties. Was sent for in the evening. Previous to that, had been spoken to by Mr. Wallington, as to witnessing his will. He said he had received instructions to make Mr. Roberts's will, and he wished me to witness it. Communicated to Mr. Wallington his view of Mr. Roberts's state on Friday evening. Urged him to haste, telling him that Mr. Roberts was labouring under great bodily affliction, and he could not tell how soon that might affect his mind. In the bodily state he was then in, he might be suddenly attacked by delirium without any premonitory symptoms. On the Sunday evening, when he arrived at Mr. Roberts's, he found Mr. Wallington with Mr. Roberts. Mr. Wallington said he wanted him to witness Mr. Roberts's signing his will, and he put the will in Mr. Roberts's hand. The candles were then lighted, and he held the candle while Mr. Roberts read the will. As soon as he had read it, he got up and went to the table, and signed it. He did so entirely of his own accord—voluntarily. He was not solicited either by Mr. Wallington or himself. Asked him if he knew the nature of the paper, and was satisfied with its contents. Has been called on to witness other wills, and it is a rule he makes to ask that question. The answer was, "Perfectly so." Upon that, signed his name as an attesting witness. When he went in that evening, Roberts put out his hand, and said, "How do you do?" His condition in the evening was, if anything, better than in the morning. His mind appeared perfectly clear. There was no excitement. He was perfectly clear, and there was nothing to excite a doubt in his mind that he perfectly understood what he was doing. Certainly would not have signed the will if he had had the least doubt of his capacity to execute it, or understand what he was doing. Saw Mr. Roberts again on Monday morning, and found him pretty much as he had left him the night before. In the evening of Monday, he thought him a little more excited. Had not been able, up to that time, to induce him to take any medicine. On the following day, he was more restless, and had more fever. On the Tuesday, Drs. Conolly, Jeaffreson, and Franklin met in consultation upon the case. Mr. Roberts afterwards got worse, and on the 8th or 9th was rather violent, and there was considerable delirium, requiring restraint, which subsided in the course of the day. Delirium showed itself at intervals. There were at times paroxysms of the disease, attended with delusions. He had temporary delusions and lucid intervals.* The delusions were never reproduced again in the same form. The symptoms improved after the 15th December up to Christmas, and then they became worse. He had a long interval at Christmas, when the whole body and mind were much improved. The bad symptoms re-appeared in the beginning of January. He suddenly got worse again, and continued growing worse till he died on the 1st of February, 1854. The excitement on the Saturday was the consequence of the diseased state of his liver. The same cause produced the excitement on the Monday and Friday, and so on throughout the illness. *Thought there was nothing more than a functional disturbance of the brain.*† In the earlier part of his attendance, did not consider that Mr. Roberts was suffering from

* Dr. Franklin was never asked to explain what he meant by a "lucid interval."

† Compare this statement with the actual organic changes found in the brain after death, and Drs. Franklin and Jeaffreson's medical certificate as to the cause of death, viz. "*sub-acute arachnitis of two months' duration.*"

any structural alteration of the brain; the functions of the brain were not destroyed, but disturbed. In the latter part of the disease it was very evident there was either a structural alteration or a congestion of the brain.

Dr. Conolly went to see Mr. Roberts on Tuesday, December 6th, 1853, at his house at Leamington, with Dr. Jeaffreson and Dr. Franklin. Found him walking about with his hat on, moving from room to room; often walking to the door as if with an apparent intention of walking out—then coming back again—restless. Thought him looking extremely ill. His aspect indicated the existence of some bodily complaint. His colour was bad, and he was dull, depressed. He answered distinct questions distinctly; there was no manifestation of violence, merely restlessness. He persuaded him to take some medicine. Called again on Tuesday evening. He had then become quiet. He had taken some medicine. He was confused, but he observed no delusion. On the following morning found him very remarkably improved. There was no appearance of confusion; he was quite calm, and clear, and, he might say, quite rational. Did not see him again after Wednesday morning. Wrote to Mr. Alderton (the attendant) on the day that he arrived at Leamington, seeing that Mr. Roberts was so confused, thinking that he might walk out or get into danger; and thinking that he was not fit to take care of himself, and wanted a person near him. He was also induced to do that from the information he had received, that he had been more troublesome than he was then. From the great improvement which took place between the first day and the second, he thought that medical treatment would have a great control over the mental and bodily health. Hoped with a bodily improvement the mind might be entirely restored, but he thought him seriously ill. Thought that both his mental and bodily state were seriously affected, and that he would die. With regard to the morbid appearances of the brain after death, he confessed his opinion to be, that the disease of the brain, as disclosed by the *post-mortem* examination, was not altogether of a standing so recent as the 4th of December. From what he saw of him on Tuesday, and the remarkable change which he observed in him on the Wednesday, it is clear that he might have had such distinct changes before then; and he might have them often. "It was not a slight change in the testator's state, but a very striking one—a complete change. *From the change on the Wednesday, he had no doubt that he was* FULLY COMPETENT TO MAKE HIS WILL ON THE SUNDAY, the 4th. *From what he saw of him on Tuesday only, he should have doubted that!*" We beg our readers' particular attention to this opinion.

*Dr. Jeaffreson* knew him about eight years before his death, and had professionally attended him. Considered him quick, shrewd, and intelligent. Accompanied Dr. Conolly and Dr. Franklin to see Mr. Roberts on Tuesday. His opinion agrees with Dr. Franklin as to his then state. He was extremely ill, with a great deal of febrile disturbance; very loaded tongue, and exceedingly quick pulse. He agreed in the medicine that was prescribed for him. Visited him next day. He was rather better. On the Wednesday he appeared more conscious and more disposed to enter into conversation than on the previous day. On the Wednesday his mind was sufficiently clear to enable him to understand any act of business. He got much worse after the Wednesday, subsequently to which he became calmer and better. About Christmas he seemed to be getting better. Was a party to making an arrangement as to his going away for a short time to a friend in the country. Assisted at the *post-mortem* examination. He drew up the report. Considering what was his condition on the Wednesday, there is nothing, in his opinion, inconsistent in the supposition of his being perfectly competent on the Sunday. "*A great deal would depend whether certain tests were applied to his mind on the Sunday, and on all occasions.*"

*Dr. Alfred Swan Taylor* deposed that he had heard the whole of this case. He had had his attention directed to the report of the *post-mertom* examination. His opinion upon the appearances presented by the brain, was, that they did not necessarily show a disease of long-standing. They indicated structural derangement of the brain, but not to a very great extent. They indicated structural derangement to such an extent as might have been produced within a period of four or five weeks, with the exception of the thickness of the skull, which must, of course, be of longer duration. He did not think they must have begun some months before in a slight inflammation of the membrane, until, within a month of the expiration of life, they assumed the aggravated shape in which they appeared after death. Having heard various facts spoken to connected with Mr. Roberts's illness on the Friday and Saturday, the alteration in his state on Sunday, and so on, it left him nothing to doubt as to his testamentary competence on the Sunday. Assuming all these facts as stated, assuming that on Monday he had the delusion as to the chimney being on fire, and on Tuesday that he was excited, but on Wednesday improved, and taking the rest of the case from Alderton's account of subsequent delusions, there was nothing, medically or physically, inconsistent with his being in a sane mind to make a will on the Sunday.

The following witnesses were examined on behalf of the defendant:

*Henry Barnett*, a gardener, had known him a good many years perfectly well. He was in the habit of coming to his place and stopping, and talking to him a considerable time. When he put up for Warwick he canvassed him. *When he first knew him, he was a sensible and rational man. Last year he became altered a little; he became more "blusterous." He talked more at random than he used to do.* He asked him about planting *five acres of asparagus, and, after that, proposed planting three acres of strawberries. He said he wanted to rent land at* 10*l. an acre, and pay* 50*l. per cent. on the outlay.* He recollected when Lord Warwick died. He told him he should *put up for member for the county.* He appeared to be in earnest, and asked him for a vote. All his talk was about elections and taxation; and then he was "blusterous" with his stick. He used to be a clever man; and became a more "blusterous" man than he used to be.

*Henry Needle* had known him for nearly forty years, and been in the habit of seeing him often. Last summer called upon him at Leamington. He complained that he had been ill-used at Warwick, but said he should come again. He complained that he had been charged wrongfully in money matters. *He was very much excited. He put himself into a fighting attitude, and seemed very desperate.* The language used by him was *not very much, but he seemed very agitated.* His manner at *that time was different from what he had known him before.* Thought from his manner that his mind was affected. Met him on the day of Lord Warwick's funeral (August 19). He said, "*I am coming for the county; I hav'n't given it in yet; I am coming for the county.*" Nothing else passed. His *manner in saying those words was not the same that he thought it ought to have been.* Recollected seeing him at the races in September; he had been riding about. He was on horseback, and talking in a very high sort of way. *He talked very loud, like a very great man.* Had seen him riding about very fast indeed, at many different times, up and down the streets, unnecessarily fast.

*Mr. W. Carpenter* had seen him at meetings of the Local Board in August 1853. *He was violent and loud in his language.* His tone was most violent, and his manner as though he was raving on the subject.

*Mr. H. Houghton Young:* Mr. Roberts was very violent at times at the meetings of the Local Board, especially when excited. On one or two occasions he was very much excited.

*Mr. F. Bowman:* Mr. Roberts bought a piano in November last for 175 guineas, and a week after paid for it. The next day he called again, and told him he had had a disagreement with Mrs. Roberts about the piano, and he wished him to take it back. *He was very much excited. He cried.* He offered him 20*l.* to take it back, and he agreed. He told him that Mrs. Roberts had disapproved of his buying the piano, and it had led to a serious quarrel. *He cried all throughout the last interview.*

*James Coleman:* In the autumn of last year his manner was changed. He saw him one day riding up Leam Terrace *with his white hat on his stick. He was spinning it round.* It was not a hot day. It was in the month of October.

*James Shepherd*, a porter, saw Mr. Roberts at Messrs. Cookes's workshop about three weeks or a month before he went to his house (*i. e.* before the 5th December). *He seemed in a very excited state, singing, whistling, and dancing.*

*Joseph Beeson*, a police officer, of Leamington, recollected that on *Saturday the 3rd of December last he was on duty in the Upper Parade. Mr. Roberts came up to him. He had his coat and waistcoat unbuttoned, and his slippers were down at the heels: it was very cold weather. It was about half-past four in the afternoon. He said, "Policeman, come here; I want two policemen to protect me. Dr. Franklin is going to kill me." He appeared to be very much excited, and in a deranged state of mind.* Told him one of their men had just gone towards his house, and if he wanted any more assistance he would come. Followed him to the corner of the Parade, as far as Warwick-street, when he went in the direction of his own house. There were several persons passing by, who turned round to look at him. Had seen him riding furiously up the streets. He had spoken to him, and told him if he did not desist from it, he must report him. He was in the habit of doing it when he came out on horseback. He was always fond of galloping his horse. He put on his hat, and said, "I'll be d—d if I have this." *If Mr. Roberts had not been a gentleman whom he knew, he should have taken him to the lock-up as an escaped lunatic!*

*Daniel Talbot*, another policeman, was on duty on Saturday, the 3rd of December last, in the evening, in the street near Mr. Roberts's house, and saw Mr. Roberts. He was going in the direction of his own house. *He had got his waistcoat and coat unbuttoned; and his hat in his hand, swinging it. He had slippers on which were down at the heels. He was making a noise, swearing to himself, as he went along.* Heard him damning and cursing as he went along, but he took no notice of it.

*W. Shirley Roby*, Superintendent of the Leamington Police, had often seen Mr. Roberts riding furiously through the streets, and several times in a dangerous manner. Had frequently cautioned him to ride less furiously. Had heard him cry "Tally ho!" as he was riding along the streets last autumn. Always thought there was something strange about him.

*W. Hughes Payton:* On Saturday evening, 5th December, called to inquire how Mr. Roberts was. Saw Mrs. Roberts, who appeared in great distress, and requested him to talk to her husband. They saw him pass on the other side of the street, and went over to him. *He appeared in a most excited state at that time. He did not seem to alter in consequence of his speaking to him. They could not get him in.* Mr. Cookes went and brought Mrs. Roberts. *She cried very much, and made every effort a poor woman in her distress could. She addressed him in the most endearing terms, and he would not go in for some time. He declared, over and over again, that he would not go into the house. He had frequently heard him talk about Parliament in a very excited manner.*

*John Mander* said, Dr. Franklin sent him to Mr. Roberts's house on Saturday night about ten o'clock. Dr. Franklin came to his house, and said he wanted him to go to a gentleman's house, and to get another man to go with him, and that very quietly. He got another man, and went to his house. He told him to go to Mr. Roberts's. They sat up all night, but were not called upon to do anything. Had been employed by Dr. Franklin before, to look after a woman who was mad.

*James Coleman:* On Monday, 5th December, 1853, he was directed to go to Mr. Roberts's house to render assistance. Saw Mr. Roberts, who looked very wild. He was running round the table, and a female servant was running before him. She seemed a good deal frightened. Mr. Roberts appeared very excited. He caught hold of Mrs. Roberts by the wrists: he said, "G— d—n you, fetch some brandy and water, the house is on fire; look at the flames." Was afterwards sent into the kitchen, when Dr. Franklin came. Mrs. Roberts brought down a washhand-basin with blood and water, as if somebody had been blooded.

*W. Richard Davis:* Saw him on the 5th December. There was a crowd of people round the area-gate. Mr. Roberts was trying to get out of the front door, Mrs. Roberts was trying to prevent him. He said the house was on fire. There was an ordinary fire in the grate made of Cannel coal. He said there was a hogshead of brandy under the fireplace, and the house was in flames. Stopped about an hour and a half, and tried to pacify Mr. Roberts, but could not succeed.

James Shepherd and Robert Smith, who were in attendance upon Mr. Roberts on the night of the 5th December, and subsequently, were next examined; the substance of their evidence is given in another part of this article.

*Mrs. Mary Ann Kerslake*, the sister of Mr. Roberts, deposed that she met her brother on the Emscote road, in the summer of 1853. He was on horseback. *He was riding in the most furious manner, with his arms stretched out, his feet out of the stirrups.* He passed her without speaking. On the 19th August, the day of Lord Warwick's funeral, she and her husband dined with Mr. Roberts. *She thought he was very extraordinary in his manner and conversation. He was very much excited.* She recollects, some time after that, going to his house. She was unwell, and stayed the night. When she was in bed, Mrs. Roberts came up into her room. That was on the 29th of August. He said, "Mary Ann, I am very glad to see you; I have been wishing you to come over." She said, "Your brother's going on like mad. He's been spending the money, and it will be all gone. Mr. Walter Cookes and I have been talking about what had better be done. He's quite incapable of managing his affairs, and we thought that he had better be sent away for a short time." I said, "Do not talk in that way, Elizabeth; it hurts my mind beyond everything. If it should get known in Leamington, they might put a strait-waistcoat on my poor brother. If they put him under restraint or punishment, it will be the death of me." She said, "He has actually been about purchasing an estate at Wolston, fit for a nobleman, and Mr. Walter Cookes, who has the estate for sale, has remonstrated with him; but," she added, "he has not the money to pay for it." I said, "I should fancy my poor brother must be out of his mind." I think she said, "Yes, I think he is." In the course of the conversation, she said he got up at twelve o'clock at night, and said there were men outside, bailiffs, waiting to come into the house. She remembered the Queen having passed through Leamington last autumn; she thought about the end of August. After that, Mrs. Roberts said Mr. Roberts was on the platform, and making a great noise; that Lady Dubonlay was there, and that he was talking to her ladyship in a very queer way, and said, "If your ladyship wants any sum of money, I can lend it to you. If your ladyship wants a thousand pounds or upwards, I can lend it to you. I shall be Chancellor of the Exchequer soon." I don't know that she said he said so to Lady Dubonlay; but he said it on the platform, and it was all part of the same conversation. She said that she felt quite ashamed of him. She said that some man who stood behind him said, "D—n you, you d—d fool, I think you will be in a madhouse before you are Chancellor of the Exchequer!" She saw her brother several times after that. She perceived that he was getting worse in his mind. Recollected, on Saturday the 3rd of December, receiving a note from Mrs. Roberts. She went over to Leamington immediately. She saw her brother there, and as she went in, he was pacing up and down the room in a very wild manner. He came close up to her, stared at her with a wild vacant stare, his eyes so large and glassy, but did not appear to know her. He did not appear conscious that any one stood before him. She said, "Henry, dear, how do you do?" He made her no answer, but soon after left the room. She was very much frightened at his appearance, and burst out crying. Mrs. Roberts came into the room, and shook her head, and seemed to intimate by that, "Poor fellow! he is out of his mind." Mrs. Roberts said, "I had a great piece of work with him yesterday; he was raving all day to come over to see you, and we could not pacify him. He wanted to come over to see you at ten o'clock at night." She said that he had not been in bed for three nights. She could perceive that he was pacing up and down the drawing-room while she had this conversation with Mrs. Roberts. She said he had been fancying, on the Friday, that the bailiffs were outside the house. She said he kept saying, "The bailiffs are outside; we must go—we must go; they wont let us have any tea." He came into the room where she was during the day. He kept running up and down the stairs. He seemed to have no particular object in doing so. He was out of her sight for a considerable time. She spoke to Mrs. Roberts about the medical advice she had. She told her that Dr. Franklin was not sufficient for such a case as her brother's; she ought to have superior advice. She suggested that Dr. Jeaffreson, or some one who understood his case better, ought to be called in, and if Mrs. Roberts would not call him in, she would. She made no remark on that. She dined there with Mrs. Griffiths. Mrs. Roberts was attending to Mr. Roberts in another room. She recollects Mrs. Roberts putting a note into her hand from Mr. Wallington, and saying, "Here, go into the other room and read that." It purported to come from Mr. Wallington to Mrs. Roberts. Could not say the date of it. The contents of it were that—"I've seen Dr. Franklin, and it is his opinion that the most energetic measures must be resorted to, to induce Mr. Roberts to make his will. It is Dr. Franklin's opinion that he cannot retain his faculties three days longer; for instance, he is now wanting to mortgage his property. You have influence, and must exert it to the utmost. Tell him that you will be getting old, and that you will have to work for your bread, and will be left penniless. Dr. Franklin and I shall be at your house in the evening, or it may be too late." It was his "faculties" which the note said he could not retain for three days, and not his "life." She had some further conversation with Mrs. Roberts. Her brother was not present. She wrung her hands in great distress, and pressed her very much to go to Mr. Smith, to get him to come and use his influence with her brother to induce him to make a will. She had not said one word before that note was put into her hand—her brother's property never crossed her mind. Having returned the note to her, Mrs. Roberts was the first who spoke about the will. She recollects some medicine being offered, and Dr. Franklin's name was mentioned. He did not consent to take it; but raved about it. He said, "I wont take that medicine—I know all about that;—no Dr. Franklin shall make me take that." She left the house, she thinks, between five and six. There was nothing to fix the exact hour. Did not go over on the Sunday, being ill in bed from seeing the state in which her brother was. She went over on the Monday morning. Heard that men had been called in. Mrs. Roberts said Mr. Wallington had been with her brother, and came out of the room with a paper in his hand, and said, "It is done," in a most emphatic manner. She stopped there on the Monday night. His sleeping-room was not far from her bed-room. He was in the drawing-room all night, and her bed-room was over it. She heard a great noise in the night. It was a dreadful moaning noise. She could hear the voice of a man and a female. She thought that the male voice was that of her poor brother. She is of opinion that he was up the greater part of the night. Mrs. Roberts had the greatest difficulty in getting him across the hall to bed; so the servants said. She was there at the time the three doctors arrived, on the Tuesday. Mrs. Roberts made an objection to her staying. She was not allowed to see her brother on that occasion.

*Hannah Lucas* went into the service of Mr. Roberts, as cook, on the 17th of November, and remained till the 31st January. When she first went Mr. Roberts seemed to be in a good state of health, but he got worse after she had been there some time. She recollected the beginning of December. He was then very excitable. She did not see much of him, her occupation being in the kitchen. She believes, on *Thursday, the 1st of December, Mrs. Roberts came into the kitchen, and said, Mr. Roberts had been in a very excitable state all night. She said he thought there were men opposite his window who wanted to break into the house; and that he had gone into the drawing-room and procured some papers, and destroyed them in his bed-room.* That was before the men came on Saturday night—a few days before. On the Friday morning, Mr. Roberts was in an excited state, walking by the kitchen door. It was in the morning, after breakfast. He was walking below in the servants' hall, where some workmen were hanging bells. She saw his face, but he did not speak to her. She thought he looked rather wild. He did not come into the kitchen that day. He told the workmen to leave their work and go directly. He spoke this in a quiet manner. They had not finished. They then stopped, and left the premises. She did not hear him give any reason for discharging them. On Saturday morning, she saw Mr. Roberts between eleven and twelve. He came into the kitchen and brought a piece of meat, which he asked her to dress for dinner. He gave it to her. He spoke very calmly. He had never brought meat into the kitchen before. She believed he had let the butcher in himself. She saw no more of him that day. Mrs. Roberts asked her and Alice Steel, the housemaid, if their assistance was required that night, would they be on the alert. Nothing occurred that night. Tilsley, the gardener, was not in the habit of sleeping in the honse, but he stayed there on Saturday night. The men from Dr. Franklin came that night. Saw Mr. Roberts early on Sunday. She went into the breakfast-room to take orders for the dinner. Mr. Roberts then appeared calm and exhausted. *He was sitting, in a crouching position, in a chair. He did not speak while she was in the room, but appeared unconscious.* Between eleven and twelve, Mrs. Roberts came into the kitchen, and asked her to send Tilsley for a bladder; but did not say what was to be done with it. She went out from three to seven in the evening. She had not seen Mr. Roberts again before she went out, nor did she see him afterwards. Before she went to bed, Mrs. Roberts asked them to sleep in the coronet-bed, in a room they had not slept in before. It was on the same floor as Mrs. Roberts's room. They slept in that room, and heard nothing in the night. Saw Mr. Roberts on *Monday morning, about ten o'clock. He came into the kitchen and chased her round the kitchen several times, and called for brandy. He afterwards said the house was on fire, and I was to take him some water.* He remained in the kitchen about five minutes. Mrs. Roberts was with him at the time. Made her escape down some steps. They called in assistance. Mr. W. Phillips was the first called in. Steel called them. Dr. Franklin had not been there that morning, but was sent for when this took place.

*Alice Steel*, the housemaid, went into Mr. Roberts's service on the 11th of November, and remained till after his death. A day or two before the Saturday, Mrs. Roberts said Mr. Roberts had got up in the night and burned some papers; that he fancied he saw men opposite the house who wanted to take his life. She told her he had struck a light behind the curtain, because he was afraid they would see him on the opposite side of the road. On the Friday, Mr. Roberts wished to see his sister very much, and Mrs. Roberts said she was afraid she must send for her, or else Mr. Roberts would go over to see her. He was not very excited that day. On the Saturday evening, Mr. Roberts went out with his sister; he afterwards came back, and was outside the door, and Mrs. Roberts went out to entice him. There were two other gentlemen there also. Mr. Roberts had his great-coat on, but both that and his waistcoat were unbuttoned. When he came in Dr. Franklin came, and he refused to take the medicine which he wished to be taken. He said Dr. Franklin wished to take away his life by giving him medicine which he would not take. Saw him about ten on the Sunday morning. He appeared in a very low and desponding state. *Saw him several times that day, and he appeared in the same low and desponding state. He was very quiet. She did not hear him speak all day.* He was lying on the sofa. He did not get up whilst she was there. They had tea about half-past five. She saw him about half-past seven in the evening. She took up a bladder with some vinegar-and-water. The vinegar-and-water were not used before seven o'clock on Sunday. At two o'clock on Sunday he appeared to be in a very low and desponding state. She remained at home all Sunday.

*William Tilsley*, the gardener, remembered Mr. Roberts meeting with an acccident up the Radford-road, and his head being cut; he thinks it was in September. Saw his forehead was cut. Was asked by Mrs. Roberts to sleep in the house on Friday, 2nd December. He had not slept there before. She said she had turned her servant away for misconduct, and she wished him to abide in the house if he were wanted. *There were two other men sleeping there.* Slept in the house on Saturday night. On Sunday, Mr. Roberts did not appear to be in any different state than since he had been ill. *He appeared to be more like a man that had had a little drop of grog than a person who was ill.** He could not say he was insane; he was in his usual state.

*Mr. Morris* saw Mr. Roberts on Sunday, and found him sitting, with linen cloths to his head, with vinegar and water. He was much depressed, and complained of being unwell, and seemed in much pain. He seemed, when he first entered, somewhat delirious from the pain. In his opinion the excitement under which he saw him was the result of pain.

*Dr. Forbes Winslow* was examined by Mr. Sergeant Miller, and said: I have read the account drawn up by Drs. Jeaffreson and Franklin, of the *post-mortem* examination of Mr. Roberts, and have well considered its details. I am satisfied that the structural changes there described must have been of some months', if not of longer, duration. If I had seen the morbid appearances described by these physicians, I should have had no difficulty in predicating that the person whose brain was so altered in its structure, must have, during life, manifested a disordered state of mind: this derangement may, and probably did, exist for some time prior to death. Considering the *post-mortem* account, I entertain no doubt that the structural alterations there described must have been progressing for some period,—certainly for months, and probably for years. Such a condition of brain would perhaps, in the first instance, give rise to eccentricity of conduct and irregularity of thought, which might escape observation until the disease of the brain and consequent disorder of the mind reached a further stage; and then obvious and unmistakable symptoms of insanity would be manifested. Diseases of the brain, as a general principle, are of slow and almost imperceptible growth. Referring more particularly to the account of the *post-mortem* examination now before me, I observe it recorded, that "the cranium was exceedingly thick." I do not attach much importance to that fact; for although such a condition of the bones of the skull is one of the recognised symptoms of long-continued cerebral disease and chronic insanity, it may exist, as a normal condition, without disease of the brain or insanity. It is notorious that men of great ability have had thick skulls; such was the case with Professor Porson. Looking at this symptom alone, I would attach no special value to it; but I think it assumes importance when viewed in association with the other brain conditions described in the *post-mortem* examination. The attachment of the "dura mater" to the skull in the "mesial line" is said to have been very firm. This

* In many cases of insanity, particularly in the incipient stage, the patient appears like a man under the influence of stimulants. Hence the common remark is, "he is either *drunk* or mad."

is a morbid appearance frequently discovered in cases of chronic insanity. Again, "the pia mater was found to be exceedingly vascular." This is an important symptom, as this membrane immediately invests the brain, and dips down between its convolutions. A highly congested and vascular state of the pia mater could not have existed without considerably disordering the functions of the brain. But I attach more weight to the next morbid appearance described in the *post-mortem* account. I find it stated that "the arachnoid membrane was universally distended by a large amount of serum effused underneath it; the membrane itself presenting in many parts the appearance of being somewhat thickened; and in almost all, of being more opaque than natural. At the base of the brain the sub-arachnoid effusion was, if anything, even more abundant than on the upper parts." There are no morbid appearances of the brain more generally discovered after death in cases of insanity, and insanity, too, of some duration, than such a state of the arachnoid membrane and "sub-arachnoid effusion." Such a condition, in my opinion, is incompatible with sanity. If I had examined Mr. Roberts's brain, and had known nothing of the state of his mind prior to death, I should have concluded, after having detected the appearances detailed by Drs. Jeaffreson and Franklin, that he had died from an attack of insanity extending over many months.

*Sergeant Miller:* I have in my hand the last edition of Professor Taylor's "Medical Jurisprudence." I find in it the following passage: "In some cases a medical practitioner may be required to state whether certain appearances found in the brain of a deceased person do or do not indicate the past existence of a certain degree of insanity or imbecility. *The appearances commonly met with on inspection are: thickening of the bones of the skull; close adhesion of the dura mater to the skull; great congestion of the pia mater; and opacity and thickening of the arachnoid membrane.*" Do you agree in the opinion thus expressed?

*Dr. Winslow:* Yes, I find the appearances of Mr. Roberts's brain, described by Drs. Jeaffreson and Franklin, in phraseology exactly similar to that used by Professor Taylor.

In answer to other questions, Dr. Winslow said, "as Mr. Roberts's mind must have been affected for some months, it would be difficult to describe where eccentricity ended and insanity commenced. It would be impossible, judging from the alterations found in his brain after death, without evidence as to his state of mind, to give any satisfactory opinion as to the period when he was reduced to such a state as to be incapable of doing a rational act. I consider that much of the eccentricity and oddity described by the witnesses, and which were evidently changes from his natural mode of thinking and acting, to have been the effects of incipient disease on the brain. The commencement of attacks of insanity and brain disease may be traced back, in many instances, for some years. When positive disease of the brain and obvious insanity manifests itself, and we examine the past history of the case, with the view of tracing it to its incipient stage, we often are able to detect well marked symptoms of mental disease, manifesting itself in the conduct and thoughts of the party, that had entirely escaped the observation of the patient's relatives and friends. Such a state of mind might exist for a considerable period, even for years, without exciting any suspicion as to the actual condition of the mind, unless the person so affected were to be attacked by some acute bodily disease, or exposed to the influence of a severe moral shock; then, in all probability, the incipient disease of the brain and mind would reach its crisis, and positive and unequivocal insanity develop itself. The mind may be fluctuating between sanity and insanity, and in a morbid and unhealthy state, without exhibiting any obvious manifestations. In considering the value to be attached to structural alterations of the brain, it is important to make a distinction between morbid changes detected in the grey or cortical, and that which is termed the medullary or fibrous portion of the brain substance. You may have organic alterations in the interior and less important parts of the brain, without obviously affecting the mind. There may be softening, tumours, and even abscesses, existing in the white or fibrous portion of the brain, without insanity: but no serious disease, congestion, or alteration of the cortical or grey matter on the surface of the brain can be present without disturbing the operations of thought, and deranging the mind. The slightest pressure on the exterior of the brain, even to the extent of a drop of blood or effusion of a small quantity of serum, may make all the difference between the possession of reason and insanity. I refer to this well-known pathological fact, with the view of explaining why I attach so much weight to the "abundant sub-arachnoid effusion" that was discovered in Mr. Roberts's brain after death."

*The Judge:* Dr. Winslow, if you had seen Mr. Roberts's brain, I presume you could have come to a more satisfactory opinion as to the probable duration of the disease?

*Dr. Winslow:* Certainly. Having heard Mr. Roberts described as a man of determined will and of much vigour of mind, I am of opinion that, coupled with the other symptoms of his case, the fact of his crying when he went to the pianoforte-maker was a sign that the mind was not then in a healthy condition. In insanity there is often alternately fits of excitement and depression. In incipient insanity, depression is frequently the result of bodily disease. During attacks of the acute forms of insanity, the patient occasionally exhibits transient moments of apparent calmness and lucidity, during which he is often able to recognise his own morbid state of mind, may appear to talk coherently and rationally on some trivial and unimportant points, and yet the disease of the mind be continuous. I have often had under my care cases of the kind.

In answer to a question from the Judge, Dr. Winslow said that he did not agree with the other medical witnesses, that the state of Mr. Roberts's mind entirely arose from the condition of the liver. The disease of the liver might have been the primary affection, the brain, from sympathy with that organ, being secondarily implicated; but, whether the disease of the brain was primary or secondary, the results were, according to his judgment, practically the same. I have heard detailed the symptoms manifested by the deceased on the Friday and the Saturday, and the delusions he then had, and his refusal on the Saturday to take medicine from Dr. Franklin, alleging that his life was too valuable to admit of his doing so. I have also heard it stated that on the same evening he called in a constable to protect him from Dr. Franklin, under the impression that he had designs on his life. I consider that at this period he was undoubtedly labouring under insanity. Considering his pertinaciously refusing to take the medicine from Dr. Franklin, and coupling that with the observations he made about his life being too valuable, with the fact of his not refusing to take the medicine that Dr. Conolly prescribed, I am of opinion that this was a delusion. He was evidently under the impression that he was going to be poisoned. I think there could be no doubt, from the evidence, that he was insane on the Friday and the Saturday, and that on the Monday he was unquestionably in the same condition. I am also clearly of opinion that he was in the same state on the Tuesday and on the Wednesday. I can come to no other conclusion, if any credence is to be attached to the evidence of Alderton, Shepherd, and Smith, who had the charge of him on that day, were constantly about his person, and who speak positively as to the presence of various delusions existing in his mind. I now refer particularly to Wednesday, when Dr. Conolly considered him free from all insanity. Considering his undoubted and admitted insanity on the *Friday* and *Saturday*, the 2nd and 3rd December; *his unmistakable insanity on the Monday, Tuesday, and Wednesday*, the 5th, 6th, and 7th of December, and which state continued with but slight variations up to the period of his death; bearing in mind that there was an absence of all scientific test as to the state of his intellect on the Sunday; and associating with this the serious organic changes found in his brain after death, and which must have been of some months' duration, I do not think that on the Sunday, the 4th of December, Mr. Roberts could have been of sound mind. If the insanity of the Friday and Saturday was the result of structural alterations in the brain, *those must have existed on the Sunday, in all probability affecting his mind on that day.* He might have had, on the Sunday, a temporary lull, and apparent calmness and freedom from excitement; but this condition of mind is quite consistent with unsoundness. I do not consider the symptoms those of *delirium*, but of insanity. The morbid appearances of the brain after death conclusively establish this point to my mind. If the attack had been one of *delirium* and not insanity, the state of the brain would have been very different. If, on examining the brain, Drs. Jeaffreson and Franklin had merely discovered a slight congestion of the surface, amounting to a mere blush or a fulness of the vessels, it would have somewhat altered my opinion as to the character of the mental disease. The alleged subsidence of the insanity on the Sunday is no proof of the mind being then in a sound and disposing state. A person may have an attack of organic disease of the lungs, indicated by impeded respiration, cough, purulent expectoration, fever, emaciation, &c., and all these symptoms may be considerably relieved by appropriate treatment; and, at times, the patient may appear free from serious pulmonary disease, but as long as the structural change exists in the lungs, he could not be said to have healthy organs of respiration. It is exactly so with disease of the mind the result of organic mischief in the brain; the moments of apparent calmness and rationality are illusory, the mind actually continuing, during the whole of the attack, in an unsound state. My opinion as to Mr. Roberts's unsoundness of mind on the Sunday is strengthened by the absence of all tests as to his actual state on that day. If his mind had been examined on the Sunday, with the view of ascertaining his capacity, my opinion might be modified. If I had seen him on the Sunday, for the purpose of testing the state of his mind prior to his executing a will, I should have asked him several questions as to his family, and whether there were not persons who had claims upon him. I should have ascertained if he knew the extent and nature of his property; and particularly if all the morbid delusions of the Saturday had entirely passed away from his mind. No examination of Mr. Roberts's mind on the Sunday less stringent than this would have satisfied me as to his power of disposing of his property.

*The Judge:* Do you agree with Dr. Conolly in opinion that there may be considerable structural disorganisation existing in the brain without insanity?

*Dr. Winslow:* Not without considerable qualifications. There may be structural alterations in the white or fibrous portions of the brain without producing obvious insanity; but, according to the received dicta of eminent pathologists, there can be no organic changes in the grey or cineritious parts of the brain, without affecting the operations of the mind. The grey or cortical substance is considered to be the seat of the intellect, and the source of the nervous power.

*The Judge:* Do you agree with Drs. Conolly and Taylor that it is a common symptom in attacks of acute insanity for the delusions to be fixed and permanent?

*Dr. Winslow:* I do not. In acute insanity the delusions frequently change; in chronic insanity and monomania they are generally fixed and permanent.

*Dr. Winslow*, in continuation, said: "If there had been no such evidence of serious structural disease of the brain, I should have given a much more qualified and doubtful opinion. The fact of his asking questions of friends, and conversing with them, is consistent with the continued presence of insanity. This feature is present in many cases of undoubted mental derangement."

*The Judge*, after reading the account of the disease of the liver, as stated in the *post-mortem* examination, asked the witness whether he did not consider the disease of the liver had been of long duration?

*Dr. Winslow:* Yes, for many years.

*The Judge:* Does that enable you to say the primary cause of disease of the brain was not disease of the liver?

*Dr. Winslow:* It is very difficult to tell; it is possible that the diseases of the liver and the brain may have gone on *pari passu.*

*The Judge:* Which is the most probable?

*Dr. Winslow:* I should imagine that the disease of the liver was the primary affection.

*The Judge:* Don't you conceive one of the best rules, when the question is one of degree, to look at a man's conduct and demeanour as a means of judging of his capability?

*Dr. Winslow:* Certainly.

*The Judge* then read the following portion of Dr. Taylor's evidence:—"I think that the state of the liver fully accounts for the state of the brain, the delusions being the result of delirium from bodily disease." Do you agree in that opinion?

*Dr. Winslow:* I do not.

*The Judge:* Do you agree with Drs. Taylor and Conolly, that the surface of the brain may be deranged without producing insanity?

*Dr. Winslow:* I cannot do so without throwing aside all the well established and recognised facts of pathology.

In order to complete the history of this case, we now subjoin the account drawn up by Drs. Jeaffreson and Franklin, of the *post-mortem* examination, and the medical certificate of the cause of death.

### Report of the Post-mortem Examination.

Roberts, Mr., *post-mortem* examination, Friday, nine A.M., 3rd February, 1854, thirty-nine hours after death. Body greatly emaciated. Green discoloration in parallel lines down the hypochondriac to illiac regions in either side. Cranium, exceedingly thick; attachment of dura mater to skull, in the mesial line, very firm. On removing the dura mater, the pia mater was found to be exceedingly vascular, and the arachnoid was universally distended by a large amount of serum diffused underneath it; the membrane itself presenting, in many parts, the appearance of being somewhat thickened, and, in almost all, of being more opaque than natural.

At the base of the brain, the sub-arachnoid effusion was, if anything, even more abundant than in the upper parts. In the substance of the brain, the puncta cruent were, perhaps, somewhat more numerous than normal. We were also struck by the apparently diminished proportion of the white to the cortical structure of the brain hemispheres.

The lateral ventricles did not appear to have been unnaturally disturbed, and no other morbid condition of the brain, cerebellum, or medulla oblongata was observed.

The muscles, generally, though wasted, were of a darkish colour.

The liver was found very considerably enlarged; its surface, as well as sections, very dark-coloured (more like section of spleen than liver), apparently in an advanced stage of hepatic venous congestion. The blood in this viscus was generally more fluid than might have been expected. The gall-bladder was thickened, its lining membrane curiously mottled, looking as if incrusted by yellow plates of cholesterine; though smooth to the touch, this viscus was largely distended by dark and inspissated gall, which it would have been impossible to distinguish from grumous venous blood, but for its colour in dilution with water. Ten or a dozen gall-stones, mostly very round, and of the same colour as the mottled marks on the lining membrane, were found in the gall-bladder the largest not exceeding the size of a field-pea. Kidneys, large, flabby, and rather congested; spleen, small and quite corrugated. Other abdominal viscera not examined, but presenting no external evidence of disease.

Heart, normal. Lungs, healthy, with the exception of a little emphysema of the lowermost edge on the left.

The right pleura costalis and pulmonalis very firmly adherent, from pleurisy of very old date.

(Signed) SAMUEL J. JEAFFRESON.
FRANCIS FRANKLIN.

| MEDICAL CERTIFICATE OF THE CAUSE OF DEATH. | DURATION. |
|---|---|
| Enlargement and hepatio venous congestion of liver - | Many years probably. |
| Subacute arachnitis, with serous effusion and maniacal excitement and depression of mind - - - | Two months. |

F. FRANKLIN, M.D., &c.
February 1st, 1854. SAML. J. JEAFFRESON, M.D., &c.

After a short deliberation, the jury returned an unanimous VERDICT IN FAVOUR OF THE WILL; coupled with a recommendation that 1000*l.* should be given to Mrs. Kerslake, the sister. Mr. Baron Parke concurred in this recommendation. This case excited an extraordinary degree of interest in the county, and Sir Alexander Cockburn, Her Majesty's Attorney-General, was specially retained to conduct the case for the plaintiff.

We have now submitted to our readers an impartial *résumé* of the material facts of this interesting and, in regard to its medico-legal bearings, important case. After a further and more matured consideration of the evidence adduced during the trial, we retain the opinion we then expressed, that it was reasonable to presume that Mr. Roberts was not of sound mind on the 4th of October, the day on which he executed his will. It is quite immaterial to the question really at issue, *how* he left his property, whether in the *right* or in the *wrong* channel; not that we would undervalue such evidence when questions of this nature are *sub judice*. It was apparent, that the fact of his having bequeathed his property to his widow, was considered by the jury as strong *primâ facie* evidence of disposing power on the part of the testator, and a conclusive proof that his feelings and judgment could not have been very much warped or disordered. But, in considering the case in its strictly psychological and scientific aspect, we must throw entirely aside all our natural sympathies with the widow, and consider exclusively the actual state of his intellect at the time he made a testamentary disposition of his property; in other words, the question for our consideration is: was Mr. Roberts of sound and disposing mind on Sunday, the 4th of October?

No unprejudiced person can read the evidence, particularly that relating to the *post-mortem* examination, detailed in the preceding pages, without being irresistibly drawn to the conclusion that the disease of the brain and disorder of the mind of which Mr. Roberts died on the 1st of Feb., had been for years creeping slowly and stealthily on in the delicate structure of the brain; giving rise in the first instance to waywardness of thought, acts of eccentricity, extravagances of conduct, exaltation of natural disposition and character, and ultimately terminating in confirmed insanity. It was the object of the Attorney-General to establish that the alleged mental attack was one of transient *delirium*, accompanied by lucid intervals, and not of insanity; and that the delirium was evanescent in its character, and marked by clear and undoubted intermissions. He was presumed to have had, on the Sunday, one of these returns of sanity, or lucid intervals. But Mr. Roberts's attack presented none of the well-known and easily recognised symptoms of delirium, in the right acceptation of the term. His derangement of mind was not of sudden and recent origin. It was admitted that he was a man of temperate habits, and it would appear, by the questions that were addressed to Shepherd, that an attempt was made to convey to the jury an impression that the attack was one of *delirium tremens*, or a somewhat analogous affection, and not mental derangement, according to the legal signification of the term. The medical witnesses always allude to Mr. Roberts's attack as being one of delirium; but this notion is quite inconsistent with the progress of the case, its symptoms, and the morbid appearances found in the brain after death. Dr. Franklin attributed the whole of Mr. Roberts's mental indisposition to the state of his liver. He says, "I thought the disease of the liver produced the delirium." Of course this physician considered that the organic affection of the brain had nothing whatever to do with his patient's mental malady; that it played an insignificant and secondary part in the matter; and was so trivial and unimportant in its consequences, as to merit no consideration! Without going into further detail, such was the general tendency of the evidence of Drs. Conolly, Jeaffreson, and Taylor. Dr. Conolly based his opinion of Mr. Roberts's sanity and disposing power, on the Sunday, upon his condition on the subsequent Wednesday. He saw him on Tuesday; but from his state of mind on that day, he says he should not have inferred that he was sufficiently of sound mind *then* to make his will; but finding him so composed on the Wednesday, when he next visited him, he therefore concluded *that he was of sound intellect on the Sunday!* This certainly appears a singularly illogical mode of testing the sanity of the testator! Dr. Conolly might have found Mr. Roberts of perfectly sound mind on the Wednesday, and fully competent on that day to execute a complicated will, and make a just disposition of his property; but why he should have inferred, from his apparent or evident sanity on the *Wednesday*, that Mr. Roberts was in the *same rational state on the preceding Sunday*, is, we confess, inexplicable. His sanity on the Wednesday might, even if the character of the attack had been different, be quite consistent with a wild state of mental derangement on the Sunday. Admitting Dr. Conolly to have arrived at a right opinion of Mr. Roberts's state of mind on the Wednesday, it does not in the slightest degree alter our opinion of his probable insanity on the Sunday. But let us for a moment consider what *was* Mr. Roberts's actual mental condition on the Wednesday, the day Dr. Conolly says he found him so tranquil, coherent, sane, and competent to make his will. If we are to believe the evidence of W. Alderton, J. Shepherd, and R. Smith, keepers who were employed to be with Mr. Roberts, we can come to no other conclusion than that even on *that day* he exhibited unequivocal symptoms of insanity. Alderton says that on Wednesday Mr. Roberts was in an excited state. He did not speak, but seemed as if he was going into an epileptic fit. Alderton, when he went to Mr. Roberts's house on the Wednesday, found him in charge of two attendants. It must be obvious that, whatever Drs. Conolly and Jeaffreson's opinions were as to Mr. Roberts's mental health on that day, his friends entertained a different opinion, and considered it necessary for his safety to place him under the care of two keepers! James Shepherd saw Mr. Roberts on the Wednesday, and, according to his sworn testimony, in what state of mind did he find him on that day? As his evidence is important we give it rather in detail:—

"He came again on the Wednesday evening between eight and nine. Smith had been there all day, and left when he got there. Soon after Mr. Roberts jumped out of bed, and Shepherd went round the foot to meet him. Roberts held his hands up, saying he hoped he had done nothing. Shepherd said nothing at all. He could not get him into bed again. He pulled the bell to get assistance. Roberts was in a state of nervous excitement. Alderton and Smith then came into the room, and they got him on the bed. It took Shepherd and Smith to hold him from half-past nine till twelve, when he became exhausted, and perspired very much. About half-past twelve he got quiet, and Alderton left. Smith laid down about one. Between one and two he got out and began to run round the bed on his hands and knees, crying, 'Oh! dear, oh! dear! I can't help it, I put 'em here.' 'Put what here?' I said. 'Two pegs,' he replied. I could not get him into bed, so I let him amuse himself. Smith got up between four and five, and we got him in. Mrs. Roberts came in just as we were trying to get him into bed, and touched him, saying, 'My dear, won't you get into bed.' He took no notice at first, but when he saw her he stood upright and ordered her out of the room. He considered Mr. Roberts's state to resemble that of *delirium tremens*."

Such being Shepherd's evidence of Mr. Roberts's state on Wednesday, what does Robert Smith say as to his condition on the same day? In the fore part of that day, Smith represents that Roberts talked about Prince Albert and the Queen, and said he was the only person appointed to hand the Queen from one carriage to another when she changed carriages. "When I went down to my tea," he continues, "I

left Shepherd there, and almost as soon as I got down the bell rang. I went up stairs, and found Mr. Roberts struggling with Shepherd. We got him into bed. He took two men to hold him in bed." In his cross-examination, this witness says "that on Wednesday night Mr. Roberts was very bad, and continued to get weaker, and was worse and worse during the whole time he was with him."* We have been anxious to give fully the evidence of Alderton, Shepherd, and Smith, because the whole value of Dr. Conolly's opinion rests upon his impressions as to Mr. Roberts's mental sanity on the Wednesday, for Dr. Conolly had no opportunity of seeing Mr. Roberts on the Sunday. Dr. Conolly says—

"From what I saw of Mr. Roberts on the Tuesday, and the remarkable change which I observed in him on the Wednesday, it is clear that he might have had such distinct changes before then, and he might have them after. It was not a slight change, but a very striking one; a complete change. From the change on the Wednesday I have no doubt he was fully competent to make his will on the Sunday before. From what I saw of him on the Tuesday only I should have doubted that."

In cross-examination, Dr. Conolly was asked the following question:—"From what you observed of his state on the Tuesday, from what you heard of his state on the Monday with reference to the chimney being on fire, and from what you have heard of his state on the Friday and Saturday, should you have considered that on the Tuesday he would necessarily be, or probably be, in such a state of mind as to be enabled to give instructions for conveying or disposing of real property to any one for life, and the whole, or a portion, to trustees on certain trusts, or to make complicated dispositions of his property in that way?" Dr. Conolly said,—"I think that, on the Wednesday morning, when I saw him, he was perfectly competent to do what you mention." In answer to a question from the judge as to his competency to make his will on the Sunday, Dr. Conolly said,—"It appears to me that there is nothing so difficult in what you say; but he could have understood it on the

* The same witness (Robert Smith) said, "On one occasion he (Roberts) was talking about a railway. He was calling out, "Stop her!—stop her!" The perspiration rolled down his face. I have frequently heard him talk of a ship coming in at the window, which he said was loaded with gold. He called me a thief, accused me of stealing his clothes, and said, *I made a practice of bringing women there, and keeping them all night.* He said, one morning, that he *could see people in the fire; that there was a monkey there smoking a cigar:* he said *Dr. Franklin had cut him to pieces, and took every nerve out of him; that he had taken his senses from him, and had put them into his own head, and he went about with his own senses, and his* (*Roberts's*) *too.* He objected to the name of Dr. Franklin. When alluding to his having made a will in favour of his wife, he said, "Never mind, Smith, *I shall, one of these days, lay down, and tell you I am dead; but you are not to take any notice of it." He added: "I shall order a cask of brandy, and let Mrs. Roberts have her fling at it, and I shall get my will back again. I made out that as a matter of form.*"

Wednesday, and therefore I do not see why on the Sunday his condition should not be equally good."

If we are justified in attaching any weight to the evidence of Alderton, Shepherd, and Smith, there can be no doubt of Mr. Roberts's insanity on Wednesday ; and if our readers come to this conclusion, and we cannot conceive how they can arrive at any other, the whole of the medical evidence for the plaintiff falls to the ground ! Drs. Jeaffreson and Franklin saw Mr. Roberts with Dr. Conolly on Wednesday, but Dr. Franklin, in his evidence, makes no reference to his condition on that day. Dr. Franklin says that "on Tuesday I met Drs. Conolly and Jeaffreson in consultation, at Mr. Roberts's house. He knew both Drs. Conolly and Jeaffreson well, and shook hands with them. He afterwards got worse, and on the 8th or 9th (Thursday and Friday) he was rather violent. Dr. Conolly succeeded in persuading him to take medicine on the Tuesday, which temporarily relieved him; he took the medicine in the presence of Dr. Conolly. There was considerable delirium, requiring restraint, on the 8th or 9th, which subsided in the course of the day."

Dr. Jeaffreson, when speaking of Mr. Roberts's state on the Wednesday, says:—"I visited him on Wednesday, at the request of Dr. Conolly, &c. He was rather better. He appeared more conscious and disposed to enter into conversation than on the previous day. His mind was sufficiently clear to enable him to understand any act of business. He got much worse after the Wednesday." Dr. Jeaffreson agreed with Dr. Conolly that, as Mr. Roberts was so improved on the Wednesday, there was nothing inconsistent in the supposition of his being perfectly competent to make his will on the Sunday: but he makes this important admission, "*that a great deal would depend whether certain tests were applied to his mind on the Sunday, and on all occasions.*" Now, what is this evidence worth? Dr. Franklin met Drs. Conolly and Jeaffreson in consultation on the Wednesday; and he could have given valuable testimony as to Mr. Roberts's mental condition on that day; but he appears to fight shy altogether of the subject. Dr. Jeaffreson says that on the Wednesday, Mr. Roberts was "rather better," "more conscious," "more disposed to enter into conversation than on the previous day;" but surely all these changes were compatible with considerable unsoundness of mind, rendering him quite unfit to make a disposition of his property. In the absence of all proof that these physicians tested Mr. Roberts's mind, *as it should have been tested* before they ventured to draw any conclusions as to his sanity and disposing power, our readers will agree with us in opinion that the fair presumption is, that Mr. Roberts, on the day he executed his will, was of unsound mind. If the attack of insanity, which obviously existed on the Friday and Saturday, and that had been for months progressing towards a crisis, *entirely subsided on the 4th of December*, with all its palpable manifestations that were observed on the two preceding days, only to recur again on the Monday, Tuesday, Wednesday, and until his death; if such a state of things *actually occurred*, in order to enable Mr. Roberts to execute his will in a sane state of mind on the Sunday, the insanity, we are bound to confess, was extremely facile, and very accommodating in its character!

We now proceed to lay before our readers another will case, many of its features analogous to the one previously discussed. We refer to the case of

THE DUKE OF MANCHESTER *v.* BENNETT, which was tried before Baron Parke, at the Spring Assizes, at Kingston, in the present year. The defendants were the three infant sons of Lady Olivia Ossulston, who, through their great-grandmother, the Right Hon. Lady Olivia Bernard Sparrow, endeavoured to set aside the will of the late Duchess of Manchester, made in October, 1848.

The late Duchess of Manchester was the daughter of General and Lady Olivia Sparrow, of Brampton Park, in the county of Huntingdon. By the will and codicil of her grandfather, Robert Sparrow, dated 1819 and 1820, certain estates in the county of Armagh, known as the Portadown estate, were devised to trustees to such uses as she (by her then name and description of his granddaughter, Millicent Sparrow) should by deed or will appoint, and in default of appointment, upon trust for her, to her separate use for life, and after her decease (but subject to any appointment she might make) upon trust for her right heirs. Up to the time of her marriage, which took place October 8th, 1822, this power had not been exercised, and on that occasion the Portadown estate, and other estates known as the Tandragee estate, became the subject of a settlement, dated October 3rd, 1822. By that settlement, the Tandragee estate was limited (subject to certain rent-charges therein mentioned) to trustees for a term of years, for securing pin-money to the late Duchess, and subject thereto to the use of the Duke for life, and after his death to trustees for a term of years, for raising 20,000*l.* by way of portions for younger children, and subject thereto, to such uses as the Duchess should by deed or will, and notwithstanding coverture, appoint. Power was also given to the Duke and Duchess to charge the Tandragee estate with 20,000*l.* for their own purposes; and by this settlement the Duchess exercised her power of appointment over the Portadown estate to the extent of limiting it to the use of the Duke for life. There were issue of the marriage four children, viz. Lord Mandeville, Lord Robert Montagu, Lord F. Montagu, and Lady Olivia Montagu, who afterwards married Lord Ossulston, and of which marriage the infant defendants were the issue.

It appeared that, previously to 1843, the Duchess had made two or three wills, but in that year she executed what was, in fact, her last will and testament, unless the one sought to be set aside were established. By the will of 1843, which bore date August 2nd, the Duchess created a term of 1000 years, for the purpose of raising an additional sum of 40,000*l.* for her younger children; and, after directing that the trustees should set apart a sum of 6000*l.* per annum (which by a codicil was afterwards reduced to 4000*l.*), as a fund out of which to liquidate certain charges and encumbrances on the estates, devised the estates to the use of her eldest son, Lord Mandeville, for life, with remainder to his first and other sons successively in tail male; and in default of such issue, then to the use of Lord Robert for life; then to Lord Frederick for life, with like limitations; and in default of such issue, to such uses as her daughter, Lady Olivia, should appoint, and in default of appointment, to Lady Olivia in fee; and the Duchess also directed that Lady Olivia's share in the portions to be raised should, in the event of her marrying, be settled upon her and her children, as therein mentioned. On the 3rd August, 1843, the Duchess executed a codicil, reducing the 6000*l.* per annum to be set apart to 4000*l.* This will and codicil were executed by the Duchess of her own mere motion, without any intervention on the part of the Duke.

The present issue was directed out of the Court of Chancery, for the purpose of trying the validity of the instrument made by the late Duchess, in October, 1848, the questions to be tried being, whether, at the time of the execution, the Duchess was of sound and disposing mind; whether she was aware of the contents of the instrument, and whether the same was executed by her under undue influence used over her by the Duke.

The will was as follows:—

"I, Millicent, Duchess of Manchester, wife of George Montagu, Duke of Manchester, do make this my last will and testament as follows:—I give all the real and personal estate of which, by virtue of any power, or authority, or of any separate right of property, I am competent to dispose, unto the said Duke, my husband, for his absolute use; and I direct and appoint that all real and personal estate of which I have any power of appointment or disposition, shall go to, and be held in trust for, my said husband absolutely; trusting, nevertheless, that he will carry into effect the wishes which I have expressed to him as to any general or particular disposition of any property; but this expression of my confidence shall not abridge his absolute ownership, or create any equity in favour of any of the objects of such expressed wishes; and I appoint my said husband to be executor of my will, and revoke all other wills."

We purposely abstain from taking any notice of the personalities which were imported into the case on both sides, as having nothing to do with the issue to be tried; and we shall proceed to give a brief outline of the evidence.

Sir F. Thesiger attempted to establish the validity of the will upon the following grounds: he observed that, in consequence of certain family reasons, the Duchess proposed making an alteration in her will of 1843, as she did not wish to leave her children independent of their father; and she expressed her wishes as to making such alteration to Dr. Verity, at Tunbridge Wells, in the summer of 1848. Dr. Verity told her that her intentions might be carried out by means of a power or trust, and she then said, "I must trust them all to the Duke, for if there was ever a true man, he is one." This conversation was afterwards communicated to the Duke, but he did not take any steps in consequence of it, till the Duchess spoke to him on the subject during her illness, as he said he observed there was something pressing upon her mind, when the following conversation occurred. The Duchess said, addressing the Duke, "Mandy" (short for Mandeville), "I do not like my will at all, or to leave any of my children independent of you." Whereupon the Duke said, "Would you like a will to be made, bearing on the face of it the carrying out of your wishes?" To this the Duchess assented. Mr. Beauford, the steward, was ordered to get such a will prepared, and Mr. Pearce, a most respectable solicitor, was applied to, and he made a draft will, with blanks for the names, which were afterwards therein inserted. We will not stop to inquire why it was that the Duke's own solicitor, who lived only sixteen miles off, was not applied to, nor to the hurry there was when interests of such magnitude were to be disposed of, which occasioned some severe comments from counsel at the trial. But to proceed: the will was dated October 26th, but Mr. Pearce proved that Mr. Beauford did not call on him till October 27th, and it therefore (as proved by the evidence) could not have been signed till October 28th.

It was executed in the following manner. The Duke asked Dr. Verity whether he considered the Duchess to be in a fit state to transact business, whereupon Dr. Verity went to her room, and said to her that "there was a little business to transact, if her grace felt equal to it." She answered, "Oh, the will, I suppose;" and desired to sign it at once. Dr. Verity then retired, and returned to the room with the Duke and Mr. Beauford: the Duke, having introduced Mr. Beauford, withdrew. Faravel, the lady's-maid, was in an adjoining room, the door being open, so that she could hear the scratching of the pen, and the sound of voices. The Duchess shook hands with Mr. Beauford, and asked after Mrs. Beauford and the children. Mr. Beauford then read the will aloud, and asked the Duchess whether she required it to be explained? she answered, that it had been read over to her on the previous day, and she proposed to execute it. The Duchess then signed it, she being in a recumbent position; and, as Dr. Verity said, "her hand shook, and she said to him, 'I think you must help me.'" Dr. Verity then steadied her hand, and slightly raised her, and she a little raised herself, and said, of her own accord, "I deliver this as my act and deed." The will was then attested by Dr. Verity and Mr. Beauford, and Dr. Verity said, on leaving the room (as asserted by Faravel, but denied by himself), "Now, Faravel, you must say nothing about this." In the afternoon of the same day, the Duchess took Dr. Verity's hand, and said that "she hoped God would bless what she had done."

Dr. Verity stated that the Duchess gradually recovered from the attack of October 1st, and was at length able to converse on any subject which he chose, or which she chose, and that the Duchess's best time was between the 21st of October and 12th of November. He further stated, that about a fortnight before her death, which took place on the 21st of November, 1848, she addressed her children for about a quarter of an hour, in an exhortation to do good, in such a manner that it could only have emanated from a sound mind, guided by Christian principles. The Duke also stated that he was in the habit of reading the Scriptures and prayers and hymns to the Duchess, and that she appeared to be perfectly conscious of the solemnity of the service.

The following was the theory of the Duchess's disease, as given by Sir F. Thesiger:—

The Duchess was attacked, on Sept. 12th, with hysteria, accompanied with strong convulsions; that she recovered from this attack, and was again seized with convulsions on October 1st. He admitted that she laboured under acute mania, followed by inflammation of the brain, and that unsoundness of mind existed for some days after the attack; that ulceration of the bowel, with diarrhœa, supervened, which relieved the brain, so that, as the one disease increased in severity, the other became mitigated, and terminated fatally in mortification of the bowel. He also considered that the delusions under which she laboured were the effect of opium, which was prescribed to allay the symptoms, and that they were not the result of disease of the brain.

The case of the Attorney-General, in opposition to the validity of the will, rested partly upon facts relative to the disease under which the Duchess laboured; and partly upon the opinions of medical men upon such facts.

Mrs. Kerr, the nurse who attended the Duchess, said that she, the Duchess, was attacked at Brompton with epileptic fits, on Sept. 12th; and that she was much convulsed, and remained unconscious for three days; that she was removed home to Kimbolton on September 26th; and that on October 1st she was again seized with epilepsy of a much more violent character; that the fits lasted several days; that there was striving and struggling, so that she was obliged to be held, and that the violence was so great that the parts of the body where she was restrained were observed to be black after the paroxysm had subsided; that she raved for days incessantly; that she then became unconscious, and lay quiet; that she gradually got better; that sometimes she would be rational for half an hour together, at other times she would not know what she had done half an hour before. That she had various delusions: upon one occasion she said (speaking to the Queen as if present), "Does it not occur to your Majesty that the Duchess of Manchester is in the room?" She said this on or about October 21st. She also laboured under the delusion that she was pregnant, and spoke to the nurse as if she were a midwife. She ordered baby-linen, and nursed the pillow as if it were a child. This delusion came on after the fits subsided, and lasted to within a fortnight of her death. She was directed by Mr. Hurst, the medical man, not to undeceive her as to the baby, as the Duchess took more nourishment in consequence of the idea that she would be better able to nurse the child. That she laboured under many other delusions, and was never free from them, during the whole of the illness, for twenty-four hours together. That the fits recurred from time to time. That the memory was much affected; and at times she did not know where she was, nor where she had been. That she had sores on the calves of her legs, back, and stomach, and an abscess on the side and shoulder; but that she did not complain of pain.

Faravel, the lady's-maid, stated that the fits came on with great "fixity;" that the Duchess foamed at the mouth; that the muscles of the face were drawn and convulsed, and that she afterwards became insensible. That sometimes the Duchess would be sensible for half an hour or an hour at a time; but that, up to the time of her death, she was never twenty-four hours free from wandering of the mind.

The Duchess had many visions after the second attack, and very often had delusions. She said upon one occasion, that she had been in the presence of God and the angels. Upon another occasion she fancied the house was on fire. Sometimes she fancied that she, Faravel, was a man, and that she had a coat and trousers on. At other times she said that she (Faravel) was in the familyway. Once she thought she was in a beautiful garden, which she described. These delusions would come on all at once, without warning, day and night. The Duchess was very violent after the second attack (October 1st), and could not be kept in bed. Her screams were heard in the middle of the park. Her memory was much affected. At Brompton she forgot that she had been in London; at Kimbolton, that she had been at Brompton. On the day she signed the will she was quiet, but very weak; and she laughed when Dr. Verity and Mr. Beauford left the room.

Lord Robert Montagu said, that one day, after the attack at Kimbolton, when he was reading Shelley's "Prometheus Unbound" to his mother, that she suddenly said, "Is it true that Mandeville has made a low marriage, and has got a family?" Upon another occasion she asked him whether he had married the housemaid.

We will now consider the medical evidence. Dr. Verity and Mr. Hurst were subpœnaed by the party who supported the validity of the will; Dr. Evanson, Dr. Mayo, Dr. Conolly, and Dr. Sutherland were subpœnaed by the opposite side, and Dr. Meryon was subpœnaed by both parties.

We may premise that the Duchess of Manchester's health had for many years been delicate, and she had been recommended to pass the winter in a warmer climate, on account of disease of the lungs. In 1836 she was attended by Dr. Verity, at Nice, where she had an attack of an epileptic character. In 1844, we have the evidence of Dr. Evanson, who wrote several letters, in the months of August, September, and October of that year, to Lady Olivia Sparrow, respecting the illness under which the Duchess then laboured, and from which it appeared that she was attacked with influenza, accompanied with pleuritis and pneumonia, and succeeded by fever, with congestion of the brain, the symptoms being confusion of thought, numbness of the hands, and affection of vision, for which active medical treatment, with blisters to the head, was had recourse to. This attack left the Duchess a confirmed invalid, so that she required constant medical supervision during the remainder of her life. In 1848, Dr. Verity stated that he saw the Duchess at Tunbridge Wells, where she remained about a month, and that she was then suffering from gastric derangement, and he attributed her illness to the misconduct of one of her sons. She returned to London, and was seen by Dr. Meryon, who found her suffering from dyspepsia, and then she proceeded to Brampton, the residence of her mother, and while there, on Sept. 12th, was attacked, according to Dr. Verity, with hysteria, the functions of the brain being in abeyance for several hours, and, according to Mrs. Kerr and Faravel (as we have already stated), with fits, accompanied with foaming at the mouth, convulsions of the muscles of the face and body, followed by insensibility of three days' duration.

On September 26th, she was removed home to Kimbolton, and on October 1st, she was again attacked with epilepsy. Mr. Hurst, who saw her on that day, and who slept in the house every night till October 29th (the day after the will is supposed to have been signed), said that he found the Duchess extremely excited; that convulsions succeeded each other in rapid succession, and continued during the night, followed by complete insensibility, which lasted for three days.

On October 2nd, we have the valuable testimony of Dr. Meryon, who states that he found the Duchess suffering under acute mania, with occasional paroxysms of terror and agitation, as if she were under the apprehension that some one was present who was about to injure her, as she was constantly crying out, "There he is! there he is!" Antispasmodics and sedatives were prescribed, and the patient was ordered to be kept extremely quiet.

October 3rd, Dr. Meryon thought that there were symptoms of inflammation of the brain of a mild character, which promptly yielded to the abstraction of ten ounces of blood from the arm, after which the symptoms of terror ceased.

October 4th. Hæmorrhage of the bowel occurred.

October 5th. The Duchess was slightly improved, and Dr. Meryon was able to take his leave.

October 7th. Mr. Hurst stated that he found the Duchess lying in a state of stupor.

October 9th. Dr. Meryon returned, and found his patient in a state of prostration from diarrhœa, he also found much irritation of the bowels, and he stated that he thought that the brain-affection may have been masked by the bowel-affection, and that the diarrhœa might have relieved the head symptoms, as on October 10th she was more composed. On this day paralysis of the rectum occurred, and the Duchess became unconscious of the calls of nature, and remained so to the day of her death.

On October 11th, the mind was quiet, and there was not the same disposition to ramble in conversation; the Duchess was able to answer one or two questions properly when spoken to, but he refrained from questioning her on the subject of her delusions, as he thought that he might go a step too far, and bring back all the mischief. He left Kimbolton on this day, and during the time of his attendance he considered that the Duchess was incapable of making a will, or of transacting any business.

October 12th. Mr. Hurst was called up at night, and found his patient violent and excited. She afterwards became unconscious, not in his opinion from symptoms of apoplexy, but from exhaustion.

October 13th. The excitement returned, and was as bad as ever.

October 14th. There was still great excitement and violence, with incoherence in the conversation.

Mr. Hurst stated, that this was the last day of great excitement, although it returned afterwards in a less degree. He also stated that the Duchess laboured under delusions. Upon one occasion, she fancied that the house was on fire; upon another, she thought that she had been delivered of a child. The state of her memory, in his opinion, varied; she was capable of making a trifling mental exertion, and almost invariably gave pertinent answers to his questions respecting her health. She was, however, at times reluctant to answer. He considered that voluntary motion was impaired, not lost; there was paralysis of the rectum, an ulcer on the back, and a sphacelating sore which made its way into the rectum, which, in any other person, would have occasioned considerable pain, but the Duchess did not complain of pain. She died of mortification of the bowel.

This concludes the evidence of the witnesses who had seen the Duchess during her lifetime.

Dr. Mayo, Dr. Conolly, and Dr. Sutherland, were called to give their opinion upon the evidence which they had heard in Court.

They concurred in the opinion that the attack of fever, with congestion of the brain in 1844, had left the brain liable, upon a sufficiently provoking cause, to a renewed attack.

That the symptoms detailed by the witnesses of the disease at Brampton and Kimbolton, in September and October, 1848, indicated the presence of constant disease in the brain throughout the period; they referred especially to the symptoms of impaired memory, of the insensibility of the nerves of sensation, and of the unconsciousness of the calls of nature. That paralysis of the rectum would not, taken by itself, account for the unconsciousness to the calls of nature, as, although it would prevent the power of retention, there would still be a consciousness of what was taking place, if the brain were in a healthy state.

That the insensibility to pain, considering the nature and extent of the sores, was indicative of disorder of the brain of a serious character.

They considered that the disease in the brain was the primary disorder, and that it was complicated with, and was not the result of, disease of the bowels. That the case was one of acute mania, terminating in chronic mania; and they considered that there was organic disease of the brain, from the fact of the disease being accompanied by epilepsy, from the impaired state of the memory, from the insensibility to pain, and from the unconsciousness of the calls of nature.

They considered it improbable that a person suffering under such a disease would have been able to transact any business requiring thought and reflection, or to take into consideration the circumstances connected with her property, the claims of relatives upon her, and, in short, to do anything that required a continuous process of thought and attention; and they said that they would not have witnessed a will under the circumstances, without having first tested the state of mind of the patient as to the extent to which the memory was impaired, and as to the presence or absence of delusions.

They considered that the conversation of the Duchess with Dr. Verity at the time of signing the will, was quite compatible with a state of unsoundness of mind, and that this was the reason for their considering that it was the more necessary that the mind should have been tested before the will was witnessed; for they stated that a person might be aware of the fact that a will had been read, might know that he was signing that will, and might even speak about it, and yet might have a diseased mind, with delusions lurking in it.

Dr. Conolly illustrated this fact by mentioning the case of a gentleman who would pass a whole evening in society, and make himself very agreeable in conversation, while, at the same time, there was the delusion latent in his mind that he was at the point of death, and upon taking leave of Dr. Conolly, he would always desire him to order his coffin to be ready for him next morning.*

Dr. Mayo having been asked as to the necessity of testing the mind under such circumstances, said, that he would not give much for the opinion of a medical man who, with such symptoms before him, could say that he paid no attention to the delusions.

We have confined ourselves strictly to that part of the evidence which tended to elucidate the state of mind of the Duchess at the time she made the disputed will. We have passed over those grave charges of undue influence brought against the Duke, which were not proved; and we abstain from commenting upon the conduct of the Duke as to the limitations contained in the settlement made in 1852, and as to his not having carried out the intentions of the Duchess, who only contemplated a disposition of the property in favour of her own children; and not to the heirs male of the second marriage in preference to the children of Lady Ossulston, the disputants in the present action. What we have to consider is, that part of the evidence which tends to show what the disease was under which the Duchess laboured; and whether, in consequence of such disease, her mind was so unsound as to have prevented her from making a proper disposition of her property.

We have to consider the theory of Sir F. Thesiger, who admitted the existence of acute mania during the early part of the disease, but stated that as the disorder in the bowels increased that of the brain became mitigated, so as to leave the mind in a sound state at the time when the will was made; and who attributed the delusions to the opium prescribed by the medical men.

* Compare this evidence with that given by Dr. Conolly in the preceding case of Roberts *v.* Kerslake.

We have, further, to consider the suggestion of Vice-Chancellor Page Wood, as to whether the wanderings of the mind did not partake rather of the nature of delirium than of delusion.

And lastly, we have to investigate the opinion of those who attributed the aberrations of the mind to acute mania.

First. As to Sir F. Thesiger's theory. It appeared from the evidence that an alteration in the symptoms did take place after the acute stage of the disease had passed off, the paroxysms of excitement yielded to exhaustion, the incoherent rambling to delusions which never left the Duchess's mind to the day of her death; considering the great prostration of strength from the hæmorrhage, the diarrhœa, the paroxysms of violence, and the discharge from the sores, it was not surprising that the Duchess was more quiet in the later than in the earlier stage of the illness; and it appeared that, counsel not being able to deny the fact of the existence of delusion existing daily throughout the illness, when complete insensibility and incoherence were absent, very ingeniously attributed it to the effects of opium. It was not stated in evidence in what doses the opium was administered, whether in stimulating or in sedative doses, but we have seen a copy of a letter written by Dr. Meryon to Dr. Evanson, wherein it is stated that the opium was prescribed in sedative doses with astringents, and, therefore, the stimulating effects of the drug could not have produced the delusions; but, further, the delusions were not of the character of the fleeting phantasies of the opium-eater, so that we do not consider it necessary to dwell longer upon the subject.

We pass then to the consideration of the Vice-Chancellor's suggestion. Were these wanderings of the mind the effect of fever? Dr. Meryon certainly stated that the Duchess, in the early part of October, had an attack of inflammation of the brain, but he said it was of a mild character, so much so indeed, that he only abstracted ten ounces of blood once from the arm; and this appears to have subdued the inflammatory action, which must have been very slight, for there is an absence of symptoms of encephalitis in the Duchess's case. We have no evidence of the intense, and deeply-seated pain in the head, the intolerance of light and of sound, the contracted pupils, the hard pulse, and the parched skin, so that the wanderings could not have been the effect of encephalitis; but were they of continued fever? Was the attack of 1848 a recurrence of that of 1844, in an aggravated form? We think not; for the wanderings spoken of in the evidence differ not only in degree, but also in kind, from delirium; they appeared indeed, to the medical men who saw the Duchess during her illness, and those who gave an opinion upon the evidence, to be the symptoms common to mania. Indeed Dr. Meryon was clearly of opinion that the disease was one of acute mania, supervening upon a slight attack of inflammation of the brain. We come, therefore, in the last place, to consider how far the opinion expressed by the medical men was borne out by the evidence: we do not include that of Dr. Verity, as he was placed in a peculiar position as an attesting witness to the will, and as a friend of the family.

We have seen that the brain had been seriously affected in 1844; there had, indeed, been frequent blisterings of the head, both before and after this attack, and prior to that of 1848; the brain therefore was in a state more liable to be attacked, either primarily or secondarily, by disease, than a healthy brain. We must not omit to mention that the Duchess had suffered under much anxiety of mind, on account of the misconduct of one of the members of her family; and that this produced much mental depression, we learn from the Duke, who said in his evidence that the first symptom which he observed, was a feeling of dread as to the state of her soul, and that the Duchess begged not to be left alone; this mental anxiety, coupled with the predisposition in the brain, above referred to, appears to have produced the severe attack of epilepsy on Sept. 12th, followed by the more severe one on Oct. 1st, and as so often happens, mania, accompanied by violent paroxysms, supervened. There can be little doubt what the disease in the brain was, the only doubt that can arise is, of what character the attack of mania was; whether it was continued, remitting, or intermitting; and whether, if the latter, the will was signed during a lucid interval.

There were great variations in the symptoms, and the more prominent points of the case are not difficult to discern. When the witnesses speak of convulsions and foaming at the mouth, followed by insensibility, we know what they mean; but when they come to the more delicate shades of description, and say that the Duchess gave rational answers, when she merely answered the questions put to her concerning her health, using the term rational, not as implying that the answers were an emanation from a rational mind, but merely that they were answers to the point, and were therefore mere proofs of consciousness, and of the mind not wandering at that particular moment, and upon that particular point; when also such terms are used by a foreigner (Faravel was a Swiss), it becomes the more difficult to draw a correct diagnosis between a remission and an intermission of the disease. We find that the Duchess was never twenty-four hours free from delusion, during the whole of the illness, when she was not either unconscious or incoherent, and the remissions or intermissions of the disease were stated to have lasted from half an hour to an hour; but it may well be questioned whether these intervals were not proofs of absence of excitement, and not of total absence of disease, which is the indication of a lucid interval. But this is rather a matter of curiosity for medical men to speculate upon, than for the lawyer to adduce as proof that there was, at the time of making the will, a disposing mind; for in this case, it having been demonstrated that there were delusions of daily occurrence, the burden of proof lay with those who supported the validity of the will to show that they were absent at the time that the will was signed; but this was not done, for Dr. Verity confessed that he was either ignorant of, or that he paid no attention to, the delusions; and he admitted in cross-examination that "you could not at all times have said that the mind might not have wandered if anything excited by emotion, or much mental exertion."

The Jury found a verdict for the plaintiff, thus establishing the will; but the Attorney-General on the 9th of June, moved for a new trial, on account of misdirection of the Judge, and because the verdict of the jury was against the evidence.

The Vice-Chancellor granted a new trial, but we understand that the matter has recently been compromised by the Attorney-General and Sir F. Thesiger.

We now proceed to a consideration of the case of Mrs. Brough, who was tried at Guildford, for the murder of her six children. The subjoined is the evidence adduced during the painful investigation:—

*Henry Woolgar*: I am a labouring man, and reside at Esher. On Saturday morning, the 10th of June, about a quarter to six o'clock, I was passing the prisoner's cottage, when I saw a pillow, covered with blood, hanging from the window of one of the rooms. A man named Peastly came up, and he rang the bell of the cottage. No answer was given, but I fancied I saw a shadow of some person moving in the house. I got a ladder and placed it against the window, and ascended it, and looked into the room. I then saw the prisoner standing at the top of the staircase, and I saw that her throat was cut, and her hands and face were covered with blood, and her hair hung about her face. She was making a whistling noise, apparently from the wound. I descended the ladder and went for a doctor, and when I returned I saw the prisoner lying on a bed in the house. The prisoner appeared to be waving a towel or a cloth in her hand when I first saw her, and she seemed to desire to obtain some assistance. The prisoner knew me by name, and I recognised her, although she was so much disfigured. The blood was spurting from her throat. I cannot say whether the whistling sound was caused by her endeavour to speak. The window where the pillow was placed was the one that a person in the cottage would come to who wanted assistance from the public road. I heard a noise in the house as though some person was walking about down below, and when I

ascended the ladder the person came upstairs close to the window. I have freqently seen the prisoner with her children, and she always appeared to be very good and kind to them.

*John Crockford* said: I lived about twenty yards from the prisoner's house, and I was in my garden on the morning in question. In consequence of something I heard, I went to the cottage and ascended the ladder the last witness had placed there. I saw the prisoner lying on the bed, and I got in at the window and saw one child (William) lying on the ground with his throat cut. In another room I saw two other children with their throats also cut. The prisoner was lying on a bed in the same room. Upon going down stairs I found the front door half open. In another room I found three other children, all with their throats cut and quite dead. While the prisoner was on the bed she moved her hand and nodded her head, but she did not attempt to speak. Several other persons were in the house when I went in. The first child I saw was lying in bed in a little side room. He was dressed in his night clothes. In the room where the prisoner was there were two children: they were lying on the bed in their night clothes. The prisoner was lying on the same bed and almost touching them. When she saw me at the window she nodded her head at me, and moved her hand as if asking for. assistance. The other three children were lying on one bed and undressed. I did not notice any blood on the bolt of the front door. The prisoner always seemed very kind and attentive to her children. The prisoner had a shawl over her shoulders. I cannot say whether she was dressed or not.

*William Bidser* said: I am constable of the parish of Esher, and in consequence of information I received on that morning, I went to the prisoner's house. I saw all the dead children and the prisoner. She had her night dress on. I asked her if she knew me, and she said "Yes." By the side of the bed on which she was lying there was a razor with dry blood upon it. The razor was on the same side of the bed as that on which the prisoner was lying, and it appeared to have dropped from her hand. I did not observe any clotted blood at her nostrils, but her face and breast were covered with blood. I have known the prisoner for some years, and lived about two hundred yards from her, and I considered her as good a mother as ever lived. She kept her children well dressed and clean, and acted in every way like a mother.

*Mr. Superintendent Biddlecomb* said: I went to the cottage of the prisoner on June 10th, about eleven o'clock. I had known her before. When I went in I saw a boot of a female saturated with blood, and the bolt of the front door was also bloody, apparently as if it had been drawn back by a bloody hand. Upon going up stairs I saw the dead bodies of three of the children in a small bedroom All these children had their throats cut, and the girl also had a wound on her shoulder. I found the prisoner in another bedroom. She was alone at this time, the dead bodies of the children having been removed. She was in bed and persons were attending upon her I asked the prisoner if she wished to speak to me, or if there was anything she requested, and she said, "No." I gave the necessary directions and left the house, and returned on the following day, and I was then told the prisoner wished to see me. I went to her and told her who I was, and she said she had been telling an officer all about it, thinking that she was speaking to me, but as I was come she should like to tell me all about it. I begged of her to be careful what she said, for it would be my duty to take down everything she said, and produce it in evidence against her. I cautioned her a second time, but she persisted in making a statement, which I took down in writing. On the following day I saw her again, and I told her I wished to read over to her what she had stated on the previous day, and I said I should do so steadily, and if there was anything she wished to retract, to do so. I at the same time told her that the coroner's jury would assemble that afternoon, and I should lay her statement before them. I then proceeded to read the statement to her, and when I had concluded she said it was perfectly correct, and she was prepared to sign it, and she did so in the presence of Dr. Mott, the medical attendant. She made no observation after she had signed it, except that, if she had left anything out the other officer could tell me. I took the statement originally in pencil, and it was copied afterwards in ink under my superintendence. I have not got the original, but I swear I made a verbatim copy of it. I am not aware of any one having seen the pencil writing except myself and the person who copied it.

The statement was then put in and read. It was as follows:—

STATEMENT OF THE PRISONER.

"On Friday last I was in bed all day. I wanted to see Mr. Izod. I waited all day, and wanted him to give me some medicine. In the evening I walked about, and I then put the children to bed, and tried to go to sleep in the chair down stairs. That was about nine o'clock. Georgy (meaning Georgina) kept calling for me to come to bed. They kept calling to me to bring them some barley-water, and continued calling till near twelve o'clock. Then some of them went to sleep. I could not rest. I had one candle lit on the chair. I could not see, and I went and got another candle, but still could not see. There was something like a cloud over my eyes. I thought I would go down, get a knife, and cut my own throat. I could not find my way down. I groped about in master's room for a razor. I could not find one. At last I found his keys, and then I found his razor. I went to Georgy and cut her first. I did not look at her. I then came to Carry and cut her, then to Henry, he said, 'don't mother.' I said I must, and did cut him. Then I went to Bill. He was fast asleep. I turned him over. He never woke. I served him the same. I then nearly tumbled into this room. The two children here, Harriet and George, were awake. They made no resistance at all. Harriet struggled very much after I cut her, and gurgled for some time. I then lay down and did myself. I can't tell you what occurred for some time after that, till I seemed weak, and found myself on the floor. That nasty great black cloud was gone then. Then I was thirsty, and I got the water-bottle and drank. I fell in a sitting position. I sat a little while, and got up and saw the children, and it all came to me again. I wished to call, but could not speak. I did not know what to do. I went to the window, and put something out to attract attention. I staggered back to my own bed, and lay till I heard the ringing of a bell. They made such a noise. I got up, and went on my hands and knees to the window. I could not make him understand nohow in the world. It was Henry Woolgar. I went down to unbolt the door. There was only one bolt fastened. I undid that. They can tell you the rest." The prisoner was able to articulate distinctly, with the exception of the whistling in her throat. She had a difficulty in speaking, and she was obliged to pause occasionally for breath. She was about ten minutes or a quarter of an hour making the statement. I did not put a single question to her. The whole of this statement was perfectly voluntary. Collett was the first constable to whom she made any statement. He was in attendance before the coroner, but was not examined. I am sure I took down the very words she spoke.

*Inspector Martell*, of the Surrey constabulary, said:—I took charge of the prisoner on the Sunday after the occurrence; and while I was sitting by her bedside she began to cry, and I told her not to do so, as it would hurt her. She then said, "See what I have done." I said, "What have you done?" and she replied, "You have seen it, and know all about it." She was then silent for about a quarter of an hour; and she then inquired when the jury would sit on the children, and I told her the next day. She then said to me, "Then you may tell them that I did it." I told her to remember I was not asking her any questions, and she went on to make a statement. (It was precisely to the same effect as the one made to Mr. Biddlecomb, the only additional fact being, that the prisoner said that, "if there had been forty children she should have done the same; what a pity it was she had not done herself first.") She further said, that on the morning after—she supposed she had been asleep—she for the first time knew what she had done, and added, "Oh, horrid, horrid sight!" and she went to the window and put out a pillow to try to get assistance, but no one came. After the prisoner had made this statement, she said to me, "You are Mr. Biddlecomb, are you not?" I told her I was not, and she might have observed the difference in our uniform; and she replied, that she did not pay much attention to uniform, and she supposed it did not matter. She afterwards expressed a wish to have the statement taken down in writing, but said she should like to have a sleep first. Mr. Biddlecomb arrived shortly afterwards, and I told him what had occurred. I don't know whether the prisoner had a sleep or not before she made the statement to Mr. Biddlecomb. I did not take the prisoner's statement down, but trusted entirely to my memory. I have never said before to-day that the prisoner told me to tell the coroner's jury that she had done it. I have seen a portion of my evidence in the newspapers, but it has never been given in full before to-day. The prisoner did not tell me the exact time when it occurred, but said she supposed it was about twelve o'clock at night.

*Mr. Charles Mott*, surgeon, of Walton-on-Thames, said:—I have occasionally attended the prisoner professionally, but at long intervals.

I saw her on the morning of the 10th June about seven o'clock. She was lying on a bed. There was a large incised wound in her throat, and her windpipe was about two-thirds severed. She knew me, but was unable to speak, and she nodded her head. The injury in her throat was such as might have been inflicted by the razor that has been produced. I saw the bodies of the children, and I am of opinion they must have been dead for several hours. They were nearly cold. The wound on the prisoner's throat must have been inflicted some time, as the hæmorrhage had ceased, and she had recovered from the fainting which must have followed the infliction of so serious an injury. Mr. Izod is my partner. From the position of the children, I do not think any of them moved at the time the fatal injuries were inflicted, except one.

*Peter Thomas Collett*, a police constable, said:—The prisoner was partly under my charge from the 10th to the 29th of June. I searched the house, and found a bunch of keys and an empty razor-case. On Sunday morning, the 11th, the prisoner told me that the clock would not want winding up until ten o'clock, as she had wound it up at ten o'clock the night before. On the 13th, the prisoner said she wished her daughter Mary had come, and she told me to take a box from under the bed, and I did so, and found it contained plate and jewellery. On the top of the box there was a piece of paper, and when I took this up, the prisoner said, "I thought not of doing of it until Friday night."

The paper was read; it was as follows:—"All for my daughter Mary. Her father is only seeking to get money from them as never injured him or done him any harm, so help me God.—Mary Anne Brough."

Examination continued.—On the same day the prisoner said, "This would not have happened but for my daughter and Fred. Foster. It is owing to a letter which they said they found and copied, and they took the copy to Kingston to Mr. Jemmett." The prisoner told me that this occurred three or four years ago, and Mr. Jemmett told them he could do nothing in it, as they had only got a copy of the letter. The letter, she said, was sent by a person named Woodhatch. That person has since left Esher. The prisoner also told me that Brough wanted to be parted from her. A woman, named Weller, who acted as nurse, was present when these conversations took place. She told me that this woman wanted to know the secrets of her heart, and I directed her not to put any questions to the prisoner. I put down in writing what the prisoner had said to me. (The witness handed in the paper.) The prisoner told me that if the doctor (Mr. Izod) had come, it would not have happened, and she said she wished she had taken his advice, as it would have been a great deal better for her. During the night she repeatedly asked for her children, and called out "Billy." She also asked whether it was her child that was crying. This was on the 11th of June. No child was crying when she made the inquiry, and everything was quiet. The prisoner did not say when she put the paper into the box, and all she said was, that she did not think of doing it until the Friday night.

*Sarah Weller* said:—I attended upon the prisoner while she was suffering from the injury of her throat. I took the prisoner some brandy and tea on the morning of the 10th of June, and I asked her if any of the children cried, and she said, "No, they were all asleep except the baby, and he was awake, and fetched three struggles." She then said that her husband had left her without money, and he was going to take the children from her, and she meant he should not do so. I will swear I did not put any questions to the prisoner except the one I have mentioned. I am not aware of the prisoner having complained to Collett that I wanted to get at the secrets of her heart. Collett did not caution me and tell me not to put any questions to the prisoner. It was on the Saturday morning that the prisoner said this to me. The doctor had only just sewn up her throat, but she was able to speak quite as distinctly as usual. I have never had any quarrel with the prisoner. I will not swear that she did not say to me "Get away." She had an apoplectic fit about a year and a half ago, and lost the use of one side, and since then the prisoner has not spoken so distinctly as she did before. She has constantly complained to me of her head since she had the fit, and she has told me that she felt a heaviness in her head—a "tumbling" like when she was stooping, as if she must fall, and a swimming. She had this fit after the birth of her last child. I was fetched to her one night, and I found she had suffered a great loss of blood from her nose. She appeared relieved in her head after the discharge of the blood. All this occurred before the birth of the child I have mentioned. The prisoner has suffered in the same manner since; but I have more particularly observed an alteration in her since she had the fit. I have frequently seen her laugh in a silly manner, and I observed a great alteration in her after she had the fit. The prisoner was always very kind to her children—almost too kind. She was a most indulgent mother. She has frequently complained of violent pain in her head over the eyes. I cannot say exactly when my attention was first attracted to the prisoner bleeding at the nose, but I believe it was shortly before the birth of the last child. Mr. Izod was called in to attend her, and she was ill for several days. She suffered a good deal during her last confinement. The prisoner never spoke so well after she had the fit as she did before.

*Frederick White* said:—I was at the prisoner's house on Friday afternoon, the 9th of June, to take home a tub for my uncle, who is a cooper. One of the little boys was in the garden, and he called the prisoner, and I gave her the bill for the repairs of the tub. She told me that Mr. Brough would be sure to call and pay my master, whoever he was, when he went to the mill. I did not see anything particular about her at this time.

*William Limerick*, a beerseller living near the prisoner, proved that on the same Friday morning he saw her in the garden, apparently engaged in pulling weeds. He did not see her for more than three or four seconds, and was unable to state whether there was anything extraordinary in her appearance or not. Witness always remarked that she was a very kind and affectionate mother, and her children appeared to be very fond of her. She was constantly in the habit of sending for cakes and such things for them, and he believed she sent for some biscuits for them on the day before. The children had been ill of the measles, and were scarcely recovered at this time.

*Henry Field* said he was acquainted with the prisoner's husband, and he went in the same train with the prisoner, by his direction, on the Monday before this occurrence. He saw the prisoner in London in company with a man, and on the following day he communicated what he had seen to the prisoner's husband, and accompanied him to his attorney, who gave him some advice; and, to the best of witness's knowledge, he never afterwards returned to his own house.

*John Birdsey*, a publican at Esher, deposed that on the evening of the 7th of June the prisoner's husband came to him, and he accompanied him to his own house. He rang the bell, and the prisoner looked out of the window and asked him what he wanted, and she added that she understood he was going to sleep at the "Wheat Sheaf." He said he was, but he wanted his nightcap and nightgown. Shortly afterwards the prisoner came down and put a bundle over the gate, and Mr. Brough took it up and went away.

*Annie Yates* deposed that the prisoner was her aunt, and she resided near the prisoner and her husband. They had been married nearly thirty years, and had a numerous family. Several of their children were dead. A young man named Foster "kept company" with the eldest daughter, Mary. The prisoner was forty-two years old. She said she last saw the prisoner before the occurrence on the Friday it happened. She then appeared very tired from having to sit up with her children. The prisoner repeatedly complained of her head. Three of the children were very ill with measles at the time. The prisoner frequently complained of violent heaviness in her head over the eyes, and she was relieved when she had bleeding at the nose. She appeared to suffer a great deal more after the birth of her last child. Her speech was so much affected that at times she could not speak at all.

*Mr. Izod* was then called. He said that he practised as a surgeon at Esher, and he had attended professionally upon the prisoner for several years. In 1852 she suffered from severe bleeding at the nose, and she also complained of great pain in her head, and he found it necessary to administer powerful medicines and also to blister her. In September, 1852, she was delivered of a child, and eight days afterwards she was attacked by paralysis, and completely lost the use of her left side. She also lost her speech, and her face was distorted. She gradually recovered, but never entirely regained her powers, and he observed symptoms of a disordered brain. In consequence of this, he said, he constantly advised her to avoid excitement of every description, and he felt satisfied that any sudden excitement would be dangerous to her. The witness said that he saw the prisoner on the Wednesday before the fatal occurrence, and from her appearance he was induced, then, to caution her strongly not to excite herself. He did not think it necessary to give her any medicine on this day, because there were not any new symptoms. There was always an apparent tendency of blood to the head in her.

*Dr. Forbes Winslow* was the next witness. I have carefully attended to the evidence in this case, and yesterday, also, had a long interview with the prisoner. I have heard the evidence of Mr. Izod, and it is my opinion that the attack of paralysis suffered by the prisoner was the result of a diseased brain. Paralysis may exist in some cases without actual insanity, but it is always symptomatic of a disease in the brain. Bleeding at the nose is a symptom of congested brain, and it is considered as an effort of the brain to relieve itself. During my interview with the prisoner in the gaol I did not observe any symptom of insanity. Cases of temporary insanity resulting in a desire to commit murder or suicide are very common. I have known many instances where the patient has made an attack upon some near relative with whom he had previously been on the most affectionate terms, and it frequently occurs with mothers and children. In such cases the patient suddenly suffers under a strong homicidal impulse which he cannot control; and it has happened to me to hear a patient bitterly lament being under the influence of such an impulse. The impulse is generally stronger in proportion as the parties are more nearly and dearly connected, and to the previous affection existing between them.

By the Court.—A person, whose body was fatigued by watching or exertion would be more likely to have the brain suddenly affected in this way than another; and the fact of the prisoner having been for two nights engaged in attending to her sick children very possibly rendered her mind more likely to be affected. A combination of suicidal and homicidal mania was frequently found combined, both arising from a disordered state of the brain. Witness agreed with Mr. Izod, that the condition of the prisoner's brain rendered her peculiarly liable to suffer from excitement; and he had no doubt that her brain had been in a disordered state ever since the attack of paralysis. In cases of transient insanity it was very common for patients to say that they experienced the sensation of a dark cloud passing before their eyes; and while in that condition, it was his opinion that the mind was thrown off its balance, and the patient, during the paroxysm, was not able to distinguish between right and wrong. In such a case there would not necessarily be any particular delusions.

In answer to a question put by Mr. Bodkin, Dr. Winslow expressed an opinion from what he had heard, in the prisoner's case, that her brain was structurally disorganized, and he said this would render it much more disposed to be affected by any moral shock. He went on to say that the mere fact of an enormous crime being committed without any apparent motive would not alone induce him to come to the conclusion that the party committing it was insane; but he said that if he found any one had killed a near relation without any motive, and that it appeared they had, up to the time of the act being committed, been on kind and affectionate terms, he should certainly think that, *prima facie*, it was an indication of insanity, but he should not positively come to that conclusion without regarding all the other surrounding circumstances.

Upon being re-examined, Dr. Winslow said he was of opinion that at this moment the prisoner was suffering from disease of the brain.

*Dr. Daniel* and *Dr. Ingledew* were then examined, and they stated that they concurred in the opinions expressed by Dr. Winslow.

After a masterly speech in her defence from Edwin James, Q.C., and an impartial summing up by the Judge, the jury acquitted the prisoner on the ground of insanity.

The preceding is a brief summary of the facts of this important criminal case. Abstracted altogether from the collateral circumstances surrounding the question of Mrs. Brough's alleged mental disorder, we would, *imprimis*, consider the *act itself* as symptomatic of a *questionable* if not of a *positively deranged state of mind*. We admit that evidence of this character should be cautiously received; but few conversant with the phenomena of criminal insanity, would acquiesce in the opinion that the malignity and atrocity of an act should altogether be disregarded, when estimating the state of mind accompanying its commission. Is it reasonable, for one moment, to suppose that this unhappy woman, who was admitted to have exhibited to her children, up to a short period antecedent to their death, the most kind and tender affection; nursing them with apparent motherly fondness during an attack of severe illness; sitting up with them for three consecutive nights, could, *in full possession of her senses*, have coolly murdered them, solely because she had been detected in an act of infidelity, and was in dread of being deserted by her husband! The idea is preposterous. *The act itself bears insanity stamped on its very face!*

We cannot, for a single instant, believe, that any mother, however lost to all sense of shame, and deeply steeped in vice, could, in violation of one of the most powerful instincts wisely implanted in the human heart, proceed deliberately, in defiance, and in total disregard of appeals that would have roused even the affection, and wrung the heart of a Hottentot or New Zealand savage, destroy six of her unoffending and innocent children! She says, in her voluntary confession:—

"I went to Georgy and cut her throat first. I did not look at her. I then came to Carry and cut her, then to Henry; he said, 'Don't mother.' *I said I must, and did cut him.* Then I went to Bill, he was fast asleep. I turned him over. He never woke. I served him the same. I then nearly tumbled into this room. The two children here, Harriet and George, were awake. They made no resistance at all. Harriet struggled very much after I cut her, and gurgled for some time. I then lay down and did myself. I can't tell you what occurred for some time after that till I seemed weak, and found myself on the floor. *That nasty great black cloud was gone then.* Then I was thirsty, and I got the water-bottle, and drank. I fell in a sitting position. I sat a little while and got up and saw the children, and it all came to me again. I wished to call, but could not speak. I did not know what to do. I went to the window, and put something out to attract attention. I staggered back to my own bed, and lay till I

heard the ringing of a bell. They made such a noise. I got up and went on my hands and knees to the window. I could not make him understand nohow in the world. It was Henry Woolgar. I went down to unbolt the door. There was only one bolt fastened. I undid that. They can tell you the rest."

Let the reader calmly consider the above confession, and quietly appeal to his own judgment as to the probable state of this unhappy woman's mind at the time she committed the murder. Could she, we again ask, have been otherwise than insane? "The nasty great black cloud" to which she refers was, we apprehend, one of the symptoms of that transient insanity or delirium with which we maintain she was afflicted during the enactment of the dreadful tragedy in which she played so fatal and conspicuous a part. The paroxysm of homicidal insanity was, we presume, the result of the severe mental shock to which the brain, actually disordered at the time, was exposed, when she discovered that her husband was cognisant of her guilty passion. This nervous concussion, in all probability, produced a vascular fulness, or congestion, of the vessels of the brain. She complained of head symptoms on the day prior to the murder; and, feeling the necessity of medical assistance, she sent for Mr. Izod, but, unfortunately, he did not see her. Had she, at that time, been placed under appropriate medical treatment, how different, in all probability, would have been the result? After cutting her own throat and losing a large quantity of blood, the head was immediately relieved; the overloaded vessels disgorged their contents; the balance of the cerebral circulation was established; all undue pressure on the brain was removed; and then, according to Mrs. Brough's account, "that nasty great black cloud" passed away; in other words, the mind was restored to a state of sanity and healthy consciousness. Is there anything singular or remarkable in this explanation of Mrs. Brough's condition of brain? It has frequently occurred, that an unsuccessful attempt at suicide has been made, during temporary and suddenly-developed attacks of mental derangement, dependent upon congestion of the brain, and the mind has immediately recovered its healthy condition, as the *effect* of the loss of blood following the division of some of the great vessels of the neck. This happened in the case of Sir Samuel Romily, who committed suicide during a paroxysm of mental derangement, and who recovered his senses as soon as the hæmorrhage that ensued had restored the brain to a state of healthy activity. Since writing the preceding comments upon this case, we have had kindly brought under our notice, by W. P. Stiff, Esq., one of the surgeons of the Nottingham Infirmary, a remarkable illustration of this fact. The case is one so much in point, that we make no apology for placing it before our readers.

"J. C., a clever mechanic, fifty-four years of age, was sent by the justices of the town of Nottingham to the workhouse, being found to be incapable of taking care of himself, owing to aberration of mind. On the 21st July, the day after his admission, he was found to be insane. He wandered about in a despondent manner, and laboured under several delusions; was in dread of being treated by Lynch law; imagined he had committed a robbery, and was to be hanged, murdered, guillotined, and stoned to death for it. On the 22nd, at nine A.M., one whole day only having elapsed since his admission, and when proceedings were about to be taken to give notice to the relieving officer of his unsoundness of mind, he obtained possession of a razor, and attempted suicide by cutting his throat with his left hand in a fearful manner. The right sternomastoid muscle and various vessels were divided, and the larynx and pharynx opened in the thyro-hyoid space. He lost three pints of blood instantaneously. All food taken in at the mouth was expelled through the wound. Painful cough was excited by trickling of blood into the trachea. The treatment consisted of injecting the stomach, by means of the ordinary stomach-pump, with milk, a quart at a time, and bandaging the head downwards on the chest, sutures and plasters having been found to be useless. *From the moment that he recovered from the collapse caused by the excessive hæmorrhage, every symptom of disordered intellect disappeared; he became rational and well-conducted; his despondency ceased, and his delusions vanished.* He became anxious about his recovery, submitted with eagerness to the introduction of the stomach-pump, and gradually regained his appetite and strength. On the 2nd September he was convalescent, and without having taken a single dose of medicine."

After directing special attention to the preceding illustration, we resume our analysis of Mrs. Brough's case.

After the brain was relieved, and this miserable woman was restored to a full consciousness of her awful condition, and realized in all its terrible reality the dreadful fact, that she had, in a moment of frenzy and delirium, imbued her hands in the blood of six of her children, what, we ask, was her first impulse? Did she, in conformity with what usually happens in cases of crime perpetrated in a sane condition of mind, try to conceal the fact? Did she endeavour to inculpate another in her own foul deed? Did she make an effort to evade justice by flying from the spot? Did she deny all knowledge of the murder? No! Had she committed the crime in full possession of her senses, it is reasonable and fair to presume that she would have endeavoured to conceal the fact by some kind of artifice: but she did nothing of the kind. Contemporaneously with her restoration to consciousness, as the effect of the loss of blood, arose in her mind the necessity of her adopting means for giving immediate publicity to the murder. With this object she made an effort to shout, but could not succeed in making herself heard. She was unable to utter any vocal sounds, having, in the attempt upon her own life, partially divided the windpipe. Feeling a pressing need for assistance, she seized a pillow saturated with blood, rushed with it to the front of the house, and placed it in a conspicuous position, outside of a window immediately facing the main road. So situated, it was seen by one of the witnesses, who, after giving the alarm, succeeded by means of a ladder in gaining access to the house. Need we dwell any further upon this particular feature of the case? Is the act just described consistent with the conduct of a person who had butchered her children in a condition of mind perfectly free from all suspicion of insanity? No impartial person can reply in the affirmative. We now proceed to our analysis of Mrs. Brough's alleged cerebral disease.

Mr. Izod and other witnesses speak distinctly of an hemiphlegic attack that Mrs. Brough had experienced some years previously, attended with loss of consciousness, &c. Now, from the *sudden character* of that paralytic seizure or "fit," as the witnesses described it, we have no doubt whatever of its origin or nature. It was our opinion, and we expressed it during our examination *in chief*, that the paralysis was consequent upon some slight effusion of blood, most probably in the substance of the *corpora striata* or *thalami*, that if the extravasated fluid or "clot" had been fully absorbed, the paralytic symptoms would have subsided; but finding in this case that the loss of motion partially remained, and that there existed evidently a defect in the powers of articulation, we stated, as the most probable solution that occurred to us on the moment, that this deposition, effusion, or clot of blood had not been properly absorbed, and that the brain in its neighbourhood was, in all probability, softened, or had otherwise become *permanently disorganized*. It was evident that Mr. Izod had observed a suspicious state of brain ever since the first hemiphlegic attack.

The above is the substance of our evidence, *quoad* Mrs. Brough's alleged cerebral disease. We arrived at the opinion that disease of the brain still existed, in the first place, from the *sudden* nature of her former paralytic seizure, and its association with well-marked *head* symptoms; secondly, from the partial state of paralysis *which then existed*, proving to us that some structural alterations must have occurred in the brain, as the result of her former cerebral attack. Having, as it was conceived, disease of the brain at the time, it was supposed that the mind was more liable to be thrown off its balance by any severe moral commotion or shock, than it would be if the brain was in a healthy state, and had exhibited no symptoms of prior disorder. It may be said that perfect sanity, soundness of mind, and self-control are compatible with a considerable amount of disease in those portions of the brain referred to as the probable seat of the paralytic stroke. Admitted. But in a case of this magnitude and importance, when the life of a fellow-creature is mainly dependent upon satisfactory proof being afforded of the presence of a morbid condition of brain at the time the alleged criminal act was committed, surely no reasonable person would cavil as to the exact position of the organic lesion. If the brain, the recognised organ of the mind, had undergone serious alterations in its delicate structure, who would have the boldness, when giving evidence upon oath, to circumscribe the limits of the disorganizing process, and say that the more important portions of the cerebral mass, supposed to be *directly* associated with the operations of the mind, *were not implicated.* It was sufficient for the defence of Mrs. Brough, to establish that she *had* disease of the brain. When life is suspended upon a thread, it surely would be deplorable if men of science, in recording their testimony, took into the witness-box the refinements of the schools, tried to draw nice physiological and pathological distinctions, and split hairs upon subtle points explicable only to the initiated. The presumption was, and we maintain that it was a legitimate one, that if it could be established that Mrs. Brough's brain was *actually diseased*, irrespectively altogether of its *locality*, it would be impossible to say to what extent that disorganization had proceeded.

Having sufficiently considered this division of our subject, we now proceed to view the act of homicide itself, as an indication of insanity. It has occurred to us during our practice to have been consulted by patients complaining of being subject to homicidal and suicidal impulses. They have occasionally described their state of mind in language remarkable for its similarity to that used by Mrs. Brough. Two or three singular cases of this kind have come under our observation, in which, during the height or crisis of the suicidal or homicidal delirium, patients have said, that they appeared to be in a "dreamy, misty state of mind," and surrounded by a "dark cloud." During the existence of an attack of this nature, consciousness appears to be partially suspended. The patient loses all knowledge of his personal identity, and is driven by an irresistible force to the commission of the most horrible crimes. We have known persons subject to periodical seizures of this nature, who, during the intermission, have bewailed their sad state, and have earnestly implored medical relief. In some cases the morbid desire to sacrifice human life never is absent from the patient's mind. A striking illustration of this kind we had an opportunity of examining a few months back. It was the case of a poor woman under the care of Dr. Skae, at the Morningside Asylum, Edinburgh. In this instance, the miserable woman expressed an intense and unconquerable desire to gratify her thirst for human blood. In language indicating the acutest amount of mental anguish and suffering, she bitterly lamented to us the existence of these horrible homicidal impulses. The case of Mrs. Brough presented many of the features usually found associated with attacks of homicidal insanity, as described by writers of experience, and found recorded in our recognised text-books. It is a singular, a generally admitted fact, that the suicidal and homicidal propensity frequently proceed *pari passu*. All authorities on medical jurisprudence of insanity notice this phenomenon. The same morbid state of the brain and disordered instinct that originates the suicidal impulse often develops the homicidal delirium. Mr. Sampson, in his interesting essay on Criminal Insanity, cites numerous cases of this kind.

It has been alleged that Mrs. Brough's description of her mental state at the time of the murder, and, in fact, the whole details of the sad event, were ingeniously and cunningly suggested by others, for an ulterior purpose. Such, it is evident, was not the fact. She gave the same minute account of her state of mind and the same narration of facts to the first person she saw, and with whom she had any conversation in reference to the event. Whether true or false, the history of the murder, and the particulars as to her peculiar sensations at the moment, *originated entirely with herself*. She on several occasions repeated the same facts, never once deviating in the slightest degree from the voluntary statement she gave to the Inspector Biddlecomb when he took her into custody.

In discussing the merits of this case, much stress has been laid upon the alleged immoral character of Mrs. Brough. It has been asserted that she was a depraved woman; that she was detected in an act of gross infidelity; and fearing the consequences of her vice, she, from a feeling of revenge, deliberately and premeditatively sacrificed the lives of her children, and then attempted her own! We do not deny that she was an adulteress; but if we are to form our judgment of her moral character from the evidence adduced at the trial, she is certainly not the horribly depraved woman represented by those who have severely animadverted upon her escape from the extreme penalty of the law. That her husband had good and valid reasons for suspecting her fidelity, we do not for a moment question. That, acting upon these well-founded misgivings, he employed a detective officer to watch his wife's movements, is an undisputed fact; that the man so engaged came up to London in the train with Mrs. Brough; that he was cognizant of her meeting a man at the station with whom she had evidently made an assignation, we also readily admit. *But here the sworn evidence stops.* She may, for aught we know, have proceeded onward in her career of guilt, but no evidence to justify such a conclusion was adduced in court during the trial.

The writers who have so freely commented upon this feature of the case may have been in possession of *extra* judicial information, fully warranting all their observations. But the witness only proved that he saw Mrs. Brough meet her alleged paramour at the London terminus. We direct the particular attention of our readers to this fact, for the scientific evidence could only be based upon the sworn testimony brought forward at the trial, and not upon vague aspersions and rumours suspended in the atmosphere surrounding the court. We do not maintain, that if all that has been subsequently said of Mrs. Brough's immorality had been fully established at Guildford, it would, in the slightest degree, have modified our opinion; but in justice to ourselves, and other medical witnesses engaged in the case, we are bound to direct the attention of our readers to the fact that when an *expert* is put into the witness-box to elucidate points of science, he is bound by his oath to base his conclusions solely and exclusively upon what he himself has seen, or upon the evidence he has heard adduced in court. This is in unison with the humane and just principles of British criminal jurisprudence. But yielding to our adversaries all the advantages they derive from a knowledge of Mrs. Brough's depravity, is the question at issue at all affected by it? Admit that she was discovered in the commission of an inexcusable act of gross immorality, that she was a vicious woman, had violated her marriage vow, was guilty of undeniable infidelity, how can these facts affect the question as to her state of mind when she murdered her children? To us they appear quite extraneous to the real point in dispute. It would be obviously and absurdly illogical to argue that *because* she was a debauched woman, and if sane deserved to be severely punished, that *therefore* she was in full possession of her senses when she sacrificed the lives of her unoffending offspring! Our opponents argue, in the first place, that she was not insane at all, and ought to have been hanged; secondly, that if she were insane, her mental derangement was the result of the immoral life she had led for years, and as her insanity was *self-created*, the gallows ought to have claimed her for its victim. Others have maintained, that having been detected in the commission of crime, and fearing her husband would desert her, and in all probability remove the children from her guardianship, she in a spirit of revenge murdered her children, and subsequently cut her own throat! Now, we do not deny that her insanity was the consequence of the severe moral shock acting upon a predisposed brain, and to which she was subjected when the discovery of her guilt was made. Neither do we argue that the fear of the penalties incurred by her immoral conduct had no connexion with the murder of her children. Her *morale* may have been much worse than the writers in the daily press represent; it is quite possible that her mind did brood over the misery, desolation, and utter hopelessness that darkened her prospects for the future. We admit that she felt acutely and keenly her degraded position; fully realized the fearful punishment that awaited her; was conscious that her husband would abandon her for ever, and cast her, poor wretch! upon the frigid sympathies of the world with the mark of Cain upon her forehead; that the children whom she brought into the world, and towards whom she had, as was proved by respectable witnesses, exhibited all the fond and enduring affection of a doting mother, would be taken from her. We willingly—fully admit—all these facts; but do they, we ask, establish that Mrs. Brough was, at the moment she committed the crime, in a sane state of mind, and a responsible agent? It is a lamentable fact, and the statistics recorded in the annual reports of our national Public Asylums conclusively demonstrate its truth, that much of the insanity amongst the pauper portion of our population may be clearly traced to *habits of intemperance*,—in other words, that it is *self-created.* The same cause is in operation, but to a limited extent, in the middle and upper classes of society; but insanity may be often traced to a criminal indulgence in depraved habits and vicious thoughts, to reckless and unprincipled conduct; to long indulged self-will; to a censurable neglect of the cultivation of habits of *self-control;* to an utter disregard of all mental discipline and training, and above all, to a repudiation of the principles of our holy and revered religion, and total rejection of the great scheme of man's redemption. We do not maintain that religion is, even in its most cheering and soothing aspect, invariably a safeguard and protection against so dire a calamity as the loss of reason; but we do assert that, *cæteris paribus*, the man who most fully appreciates the comforting and elevating principles of our common Christianity, and lives up to his professions, is to a great extent shielded from the influence of those causes, which so frequently derange the human mind. If we are justified in considering every person accountable and amenable to punishment whose insanity can be clearly traced *to self-created causes*, where are we to draw the line? The man who, as the result of a series of debaucheries, *voluntarily* drinks himself into a state of furious delirium, is, as long as that delirium continues, *non compos mentis*, and is not accountable, in the eye of the law, for any act committed during his paroxysm of frenzy. We may regret that there should not exist for cases like these a secondary form of punishment, which, if judiciously awarded, might prevent much of the deplorable misery we are daily compelled to witness in social life; but until our criminal code has undergone material alterations, it is not for us to draw refined distinctions, and say one class of insane persons should not escape the legal penalties to which they have by their conduct exposed themselves, simply because their mental disease can be traced to unbridled passion or unchecked vicious impulses and thoughts; and a different class of the insane, whose sad condition has originated from causes quite out of their own control should entirely escape from punishment or censure. With these general remarks, we dismiss altogether the argument of Mrs. Brough's legal accountability, based upon the presumption that her insanity was self-created, and the result of an habitual indulgence of a criminal passion.

There are a few other points of interest connected with this remarkable case, to which we, in conclusion, can only cursorily refer. We allude to the fact of Mrs. Brough's having been liable to attacks of *epistaxis*, or hæmorrhage from the nose. These discharges had been for a short time suppressed. We direct attention to this fact, not because we attach grave importance to it, but when viewed in association with other portions of Mrs. Brough's history, it is entitled to some consideration. It is quite possible that a suppression of this discharge might have created an unnatural vascular fulness, or congestion of the brain, and, with other causes, have co-operated in deranging the mind. Again, we cannot omit all allusions to Mr. Izod's important statement, that on the day preceding the murder he had noticed something peculiar about Mrs. Brough's cerebral condition, and had considered it his duty to guard her against exposing herself to causes of mental excitement. We think sufficient has been advanced to establish to the satisfaction of our readers, that if we have not succeeded in proving Mrs. Brough's positive insanity, the facts referred to demonstrate beyond all disputation that a strong *primâ facie* case was made in support of the plea urged in her defence, and that the decision of the jury was founded upon a humane, just, and enlightened consideration of the facts of this singularly painful case.

